# Supplementary material for: Dalpiciclib and pyrotinib in women with HER2-positive advanced breast cancer: a single-arm phase II trial
Source: Nat Commun. 2023 Oct 7;14:6272. doi: 10.1038/s41467-023-41955-7 (PMC10560297; doi:10.1038/s41467-023-41955-7)
Supplement: Supplementary file 1 — Supplementary Information [file 41467_2023_41955_MOESM1_ESM.pdf]

Supplementary Information

Supplement to: Min Yan, et al. Dalpiciclib and pyrotinib in women with HER2-positive advanced breast cancer: a single-arm phase II trial

Table of contents

Supplementary Figure 1. Objective response in subgroups 2

Supplementary Figure 2. Progression-free survival in subgroups of different trastuzumab-sensitive status 3

Supplementary Figure 3. Progression-free survival in patients with brain metastasis (BM) at baseline 4

Supplementary Note 1. Study protocol 5

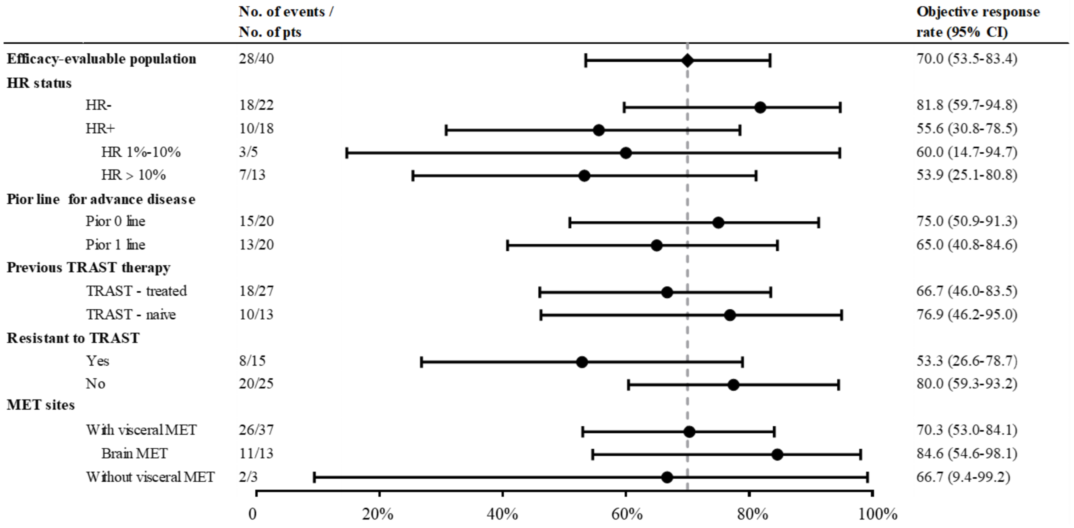

Supplementary Figure 1. Objective response in subgroups. HR- was defined as ER/PR expression in ＜1% tumor cell. HR, hormone receptor; TRAST, trastuzumab; MET, metastasis. Source data are provided as a Source Data file.

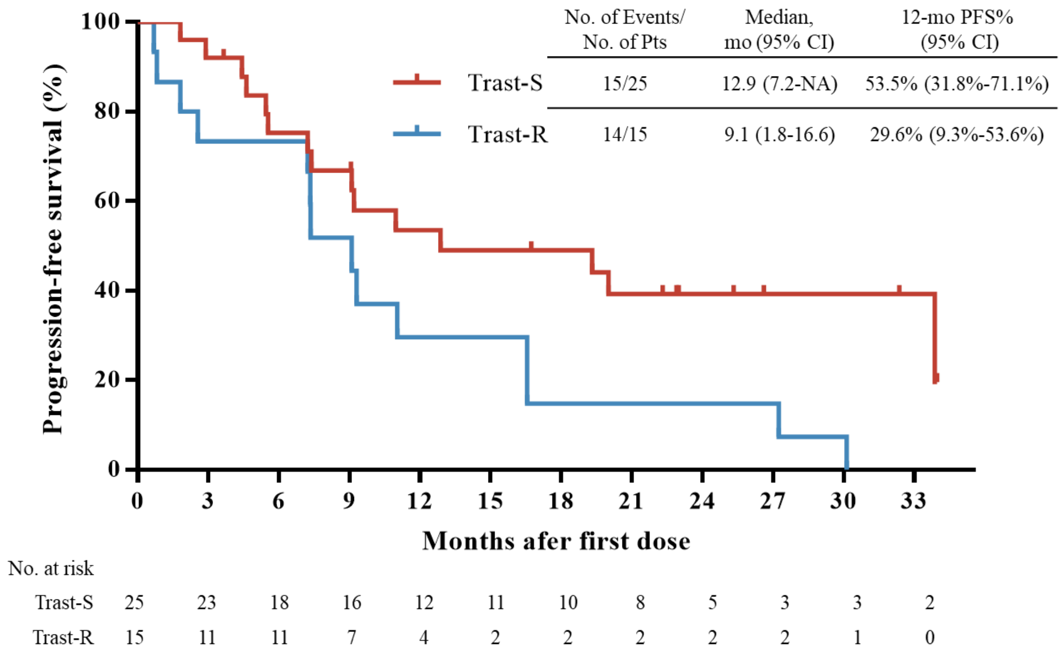

Supplementary Figure 2. Progression-free survival in subgroups of different trastuzumab-sensitive status. Trast, trastuzumab; S, sensitive; R, resistant. Source data are provided as a Source Data file.

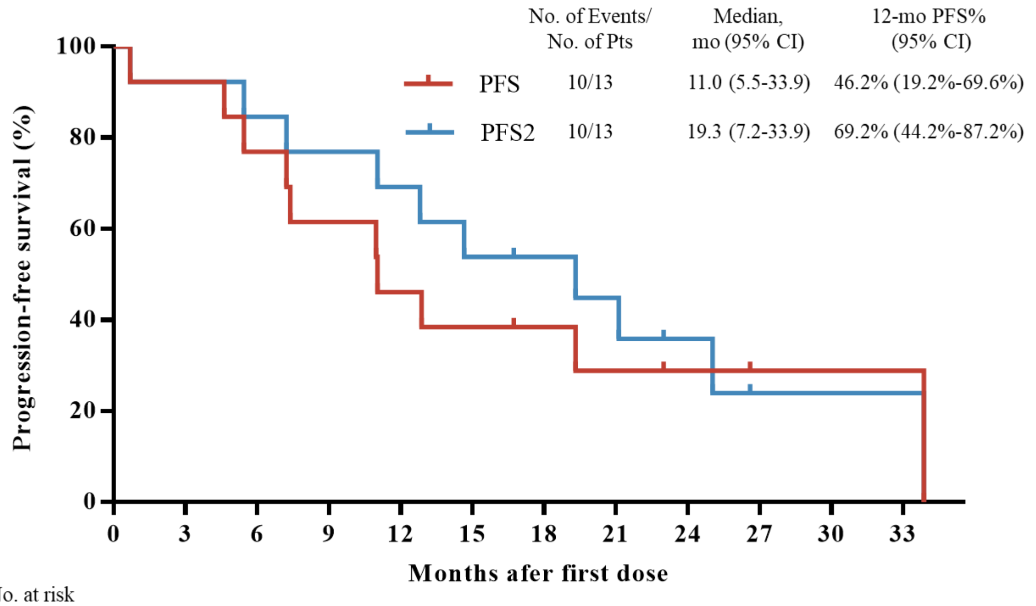

No. at risk												
PFS	13	12	10	8	6	5	4	3	2	1	1	1
PFS2	13	12	11	10	9	7	6	4	3	1	1	1

Supplementary Figure 3. Progression-free survival in patients with brain metastasis (BM) at baseline. Five BM patients progressed isolated to the brain lesions continued the study treatment after local stereotactic radiosurgery. PFS2 was defined as time from first dose of study drugs to second progression or death in BM patients. Source data are provided as a Source Data file.

Supplementary Note 1. Study protocol

For publicly posted protocols, the background is redacted because it may contain proprietary information. The appendices, generally, are not provided because they may be lengthy and contain non-essential information. The publicly posted protocol includes all the key sections that are relevant to evaluating the study, specifically those sections describing the study objectives and hypotheses, the patient inclusion and exclusion criteria, the study design and procedures, the efficacy and safety measures, and the statistical analysis plan.

An Exploratory Study to Evaluate the Efficacy and Safety of CDK4/6 Inhibitor SHR6390 Combined With Pyrotinib in the Treatment of HER2-positive Advanced Breast Cancer

Clinical Study Protocol

Scheme No.:	HR-BLTN-014
Version No.:	1.2
Version date:	September 3, 2021
Principal Investigator (PI):	Professor Min Yan
Sponsor’s Name:	Henan Cancer Hospital

Principal Investigator Signature Page

I will conscientiously perform the duties of a researcher in accordance with the Chinese GCP regulations, and personally participate in or directly guide this clinical study. I have read and confirmed this scheme (version 1.2; version date: September 3, 2021). I agree to conduct this study following this protocol and in accordance with Chinese law, the Declaration of Helsinki, and the China GCP. This research proposal cannot be implemented without the consent of the Ethics Committee, unless measures must be taken to protect the safety, rights and interests of the subjects.

Sponsor: Henan Cancer hospital

Min Yan		
**Principal investigator (Print Body)**	**Signature**	**Date (yyyy/mm/dd)**

Content

Protocol Synopsis 5
Study flow chart 9
Abbreviations 13
1 Research Background 15
2 Study Objective and Endpoints 20
2.1 Study Objective 20
2.2 Endpoints 20
3 Study Design 20
4 Selection and Withdrawal of Patients 21
4.1 Inclusion Criteria 21
4.2 Exclusion criteria 21
4.3 Treatment discontinuation Criteria 22
4.4 Criteria for exclusion from analysis 23
4.5 Trail termination criteria 23
4.6 Lifestyle requirements 23
5 Study treatment 24
5.1 Overview of study drugs 24
5.2 Dosage regimen 24
5.3 Dosage adjustment 25
5.4 Common adverse reactions and its treatment 26
5.5 Investigational products management, distribution and recycle 28
5.6 Patient compliance 28
6 Concomitant Therapy 28
6.1 Prohibited Drugs during the study 29
6.2 Drugs to be used with caution during the study 29
6.3 Drugs and treatments that can be used in combination during the study 29
6.4 Dietary requirements 30
7 Study Procedures 30
7.1 Screening 30
7.2 Trial period 32
7.3 End-of-treatment/withdrawal from study 33
7.4 Follow-up 34
8 Efficacy Assessment 35
8.1 Imaging evaluation 35
8.2 Primary endpoint 35
8.3 Secondary endpoints 36
9 Safety Assessment 36
9.1 Physical examination and vital signs 36
9.2 Laboratory test 37
9.3 ECG 37
9.4 Echocardiography 37
9.5 Adverse event (AE) 38

9.6 Serious adverse events (SAE)....39
9.7 Follow-up of AE/SAE....42
10 Clinical Trial Management....43
10.1 Ethics and Informed Consent....43
10.2 Investigational product management....43
10.3 Amendment of the plan....43
10.4 Quality management....43
10.5 Data management....44
10.6 Protocol violation....45
10.7 Data preservation....45
10.8 Publication policy....45
11 Data Analysis and Statistical Methods....45
11.1 Sample size....45
11.2 Statistical Analysis Program....46
12 Case dropout....47
13 Expected study duration....47
14 References....48
Appendix 1 Clinical Stage for Breast Cancer (AJCC TNM Staging system for Breast Cancer, 8th Edition)....
Appendix 2 patient's performance status (ECOG)....
Appendix 3 ASCO CAP 2018 HER2 Testing for Breast Cancer Guidelines....
Appendix 4 Response evaluation criteria in solid tumors....
Appendix 5 Contact for serious adverse events....

Protocol Synopsis

**Official Title**	An Exploratory Study to Evaluate the Efficacy and Safety of CDK4/6 Inhibitor SHR6390 Combined With Pyrotinib in the Treatment of HER2-positive Advanced Breast Cancer
**Sponsor**	Henan Cancer Hospital
**Principal Investigator**	Professor Min Yan
**Study Objective**	To evaluate the efficacy and safety of pyrotinib plus CDK4/6 inhibitor SHR6390 in HER2-Positive advanced breast cancer patients
**Rationale**	Cyclin D-CDK4/6-RB-E2F signaling pathway regulates the transition of cell cycle from G1 phase to S phase in breast cancer cell, thereby plays an important role in breast cancer cell proliferation. HER2 protein regulates the proliferation of breast cancer cells through signaling pathways such as PI3K/AKT, and HER2-positive breast cancer patients need anti-HER2 therapy. Patients with HER2-positive breast cancer have been shown express high levels of cyclins D1 and E1, suggesting that anti-HER2 therapy can synergize with CDK4/6 inhibitors.<br>A preclinical study showed that the combination of the CDK4/6 inhibitor SHR6390 and the anti-HER2 drug pyrotinib can effectively reduce the phosphorylation of AKT and HER3, thereby promoting the apoptosis of HER2-positive breast cancer cells and inhibiting tumors.<br>In the NA-PHER2 clinical study, palbociclib combined with trastuzumab, pertuzumab and fulvestrant can significantly inhibit Ki67 expression; a phase I clinical study of abemaciclib combined with trastuzumab compared with trastuzumab can significantly shrink HR-negative and HER2+ tumors.
**Study Population**	HER2-positive advanced breast cancer patients
**Study Design**	Single-center, open-label, single-arm
**Phase**	Exploratory phase II Study
**Sample Size**	A Simon minimax two-stage design was used to estimate the sample size. We planned to enroll 23 patients in the first stage. In the first stage, 23 subjects will be enrolled. If ≤12 subjects achieve CR/PR, the treatment was judged as invalid and the study was terminated, otherwise the study goes to the second stage, and totally 37 subjects will be enrolled. If more than 23 subjects achieve CR/PR, the treatment plan will be considered to be effective. Considering a dropout rate of 10%, totally 41 patients would be enrolled.
**Schema**	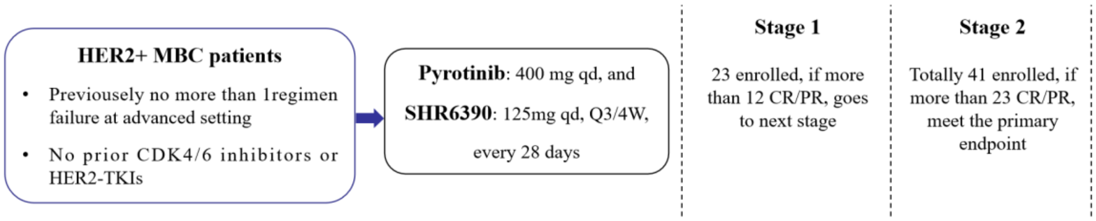
**Study Interventions**	Pyrotinib: once a day, 400 mg each time, orally administered within 30 minutes after breakfast, continuous administration for 28 days as a cycle.<br>SHR6390: once a day, 125 mg each time, orally administered for 3 weeks on and 1 week off, and 4 weeks as a cycle. It is recommended to take the medicine at about the same time every day, with warm water, and fast for at least 1

	hour before and after taking the medicine.
**Primary Endpoint**	Objective response rate (ORR) assessed by investigators based on RECIST 1.1.
**Secondary Endpoints**	Safety: ECOG performance status, vital signs, physical examination, laboratory tests (blood routine, urine routine, stool routine, blood biochemical test, coagulation test), ECG, cardiac color Doppler, and adverse events (according to NCI-CTCAE, version 5.0).<br>Effectiveness: progression-free survival (PFS) and overall survival (OS) by investigators.
**Inclusion criteria**	1. Patients with HER2-positive advanced breast cancer who had received ≤ 1 line treatment in the past;<br>2. Female ≥ 18 years and ≤ 70 years of age;<br>3. HER2-positive breast cancer is defined as immunohistochemistry (IHC) score of 3+, or IHC score 2+ and positive in situ hybridization (ISH) test (ISH amplification rate ≥2.0) confirmed by the pathology laboratory according to 2018 ASCO-CAP guidelines;<br>4. Eastern Cooperative Oncology Group(ECOG) performance status 0 to 1;<br>5. Life expectancy is not less than 12 weeks;<br>6. At least one measurable lesion according to RECIST 1.1;<br>7. Patients must have adequate organ function, criteria as follows:<br>1) Blood routine test:<br>• Absolute neutrophil count (ANC) $\geq 1.0\times10^9$/L (without growth factor within 14 days);<br>• Platelet count (PLT)$\geq 100\times10^9$/L (without corrective treatment within 7 days);<br>• Haemoglobin (Hb) ≥90 g/L (without corrective treatment within 7 days);<br>2) Blood chemistry test:<br>• Total bilirubin (TBIL) ≤1.5 times the upper limit of normal (ULN);<br>• Alanine aminotransaminase (ALT) and aspartate aminotransferase (AST) ≤3 times ULN; for patients with liver metastases, ALT and AST ≤5 times ULN;<br>• Blood urea nitrogen (BUN) and serum creatinine (Cr) ≤1.5times ULN;<br>3) Echocardiography:<br>• Left ventricular ejection fraction (LVEF) ≥ 50%;<br>4) 12-lead ECG<br>• The QT interval corrected by Fridericia's formula (QTcF) is <470 ms for females.<br>8. Patients need to voluntarily join this study after they fully understand and sign the informed consent form. Patients need to have good compliance and be willing to cooperate with follow-up.
**Exclusion criteria**	1. Clinically symptomatic patients with brain metastases;<br>2. Patients with dysphagia, chronic diarrhoea and intestinal obstruction and factors that affect the administration and absorption of the drug;<br>3. Patients who have received radiotherapy, chemotherapy, surgery (excluding local puncture) within 4 weeks before enrolment; patients who have received anti-tumor endocrine therapy during screening;<br>4. Patients who participate in clinical trials of other new drugs within 4 weeks before enrolment;<br>5. Concurrently treated, or previously treated with HER2 tyrosine kinase inhibitors (including lapatinib, neratinib, pyrotinib, etc.);<br>6. Prior any CDK4/6 inhibitors;<br>8. History of other malignant tumours within 5 years, excluding cured cervical carcinoma in situ, skin basal cell carcinoma or skin squamous cell carcinoma;<br>9. Female patients during pregnancy and lactation; fertile female patients who tested positive on a baseline

	pregnancy test; female patients of childbearing age who are unwilling to take effective contraceptive measures during the trial or within 90 days after last dose of study drug;<br>10. Allergic to the study drug; history of immunodeficiency, including HIV positive, HCV or other acquired or congenital immunodeficiency diseases, or history of organ transplantation;<br>11. Severe heart disease, including but not limited to the following:<br>• History of heart failure or systolic dysfunction (LVEF < 50%);<br>• Angina or arrhythmia of high risk or requiring treatment (eg, second-degree type 2 AV block or third-degree AV block, ventricular tachycardia);<br>• Clinically significant heart valve disease;<br>• Transmural myocardial infarction shown by ECG;<br>12. According to the judgment of the investigator, there are concomitant diseases that seriously endanger the patient's safety or affect the completion of the study (including but not limited to severe hypertension that cannot be controlled by drugs, severe diabetes, active infection, etc);<br>13. Complicated with moderate infection within 4 weeks before the first dose (eg: intravenous infusion of antibiotics, antifungal or antiviral drugs is required according to clinical diagnosis and treatment standards), or unexplained fever >38.5°C during screening/before the first dose;<br>14. Any other circumstances that are not suitable for inclusion in this study judged by investigators.
**Treatment discontinuation Criteria**	If one or more of the following conditions occur, the subject must withdraw/discontinue treatment:<br>1. Subjects withdraw their informed consent and request to withdraw;<br>2. Imaging or clinical evidence progress, unless the subject can benefit from continued treatment judged by the investigator (participants with BM at baseline getting isolated intracranial progression could continue study after brain radiotherapy till second progression if they would benefit from the treatment according to the physician discretion);<br>3. Any clinical adverse events, abnormal laboratory tests or other medical conditions that may prevent the subjects from benefiting from continued medication;<br>4. Those who seriously violated the trial protocol and the investigators assessed that the treatment should be terminated;<br>5. During the research process, the subject has a pregnancy event;<br>6. Other reasons considered by the investigator to be unable to continue the study drug treatment.
**Trail termination criteria**	Termination criteria include but are not limited to the following:<br>1. Unexpected, significant or unacceptable risks to the subject are found;<br>2. Major mistakes were found in the plan during the execution of the test;<br>3. The study drug/treatment is ineffective, or it is meaningless to continue the trial;<br>4. Completion of the trial is extremely difficult due to reasons such as severe delays in subject enrollment or frequent protocol deviations.
**Statistics**	Efficacy Analysis:<br>Point estimation will be used to assess the endpoint of the objective response rate (ORR) and 95% confidence

	intervals will be provided to represent the population. Survival analysis will be conducted using the Kaplan-Meier method, and the median of progression-free survival (PFS) and overall survival (OS), and their 95% confidence intervals representing the population will be calculated, and the survival curve will be drawn. Descriptive analysis will be used for other secondary efficacy measurements.<br>Safety Analysis:<br>The adverse events, serious adverse events and treatment-related adverse events will be analyzed mainly based on descriptive statistical methods. Abnormal laboratory tests defined as laboratory test results being normal at baseline but becoming abnormal after treatment will be included in the safety analysis.
**Efficacy assessment**	The imaging evaluation will be performed every 2 cycles (every 8 weeks ±7 days) within the first 18 cycles and every 3 cycles thereafter (every 12 weeks ±7 days) until disease progression or the initiation of new antitumor therapy in the enrolled subjects. Efficacy will be evaluated according to RECIST 1.1 criteria. Survival status will be followed every 12 weeks after disease progression or initiation of new antineoplastic therapy.

Study flow chart

	Screening period *	Treatment Course					Follow-up		
	D-28~D-1	C1			C2+	End of treatment/ exit	Safety[2]	Efficacy[23]	Survival[24]
		D7±3	D14±3	D28±3	D28±5				
Basic information									
Written informed consent	×								
Demographics[1]	×								
Medical and treatment history[2]	×								
Concomitant medications/treatments[3]	×	×							
Laboratory/Auxiliary Exam									
Blood routine[4]	×	×	×	×	×	×			
Urine routine[5]	×	At the end of every 4 cycles ±7 days				×			
Stool routine[6]	×	At the end of every 4 cycles ±7 days				×			
Blood biochemical test[7]	×		×	×	×	×			
Pregnancy test[8]	×					×			
Infectious diseases[9]	×								
12-Lead ECG[10]	×		×	×	×	×			
Echocardiography[11]	×	At the end of every 4 cycles ±7 days				×			
Clinical evaluation									
Vital sign[12]	×		×	×	×	×			
Physical examination[13]	×	At the end of every 4 cycles ±7 days				×			
ECOG PS	×		×	×	×	×			
Adverse events[14]	From informed consent to 28 days after last dose								
Efficacy endpoint evaluation									
Imaging evaluation[15]	×	At the end of every 2 cycles ±7 days for first 18 cycles, and every 3 cycles thereafter				×			
Disease progression[16]		×						×	
Death[17]		×							×
Study drug									
Pyrotinib[18]		Once daily, administrated orally within 30 minutes after meal							
SHR6390[19]		Once daily, fasting for 1 hour before or after oral administration							
Other records									

	Screening period *	Treatment Course					Follow-up		
	D-28~D-1	C1			C2+	End of treatment/ exit	Safety[2]	Efficacy[23]	Survival[24]
		D7±3	D14±3	D28±3	D28±5				
Drug dispensing[20]		×							
Drug recall[20]		×							
Subsequent anti-tumor therapy[21]							×		

Note:

The study does not allow to re-screen subjects who have failed in previous screening (that is, re-sign the informed consent and re-screening is not allowed; except for subjects who fail in previous screening due to age).

The following examinations should be completed within the time window listed in the flow chart. In case of legal holidays, the examinations could be performed ahead of schedule, and the reason for the deviation should be recorded in the eCRF. Visits are scheduled on Day 7 (±3 days), Day 14 (±3 days), Day 28 (±3 days) of the first cycle, and on Day 28 (±5 days) of every subsequent cycle. Investigators can add additional examinations or increase the visiting frequency based on the subjects' clinical condition. The results of laboratory and auxiliary examinations involved in this study can be examined by other hospitals, and it is up to the investigators to decide whether to accept the examination results.

* Screening period: physical examination, vital signs and laboratory tests (including blood routine, urine routine, liver and kidney function, blood lipids, blood electrolytes, fasting blood glucose, blood HCG, etc.) are conducted within 14 days before the start of study treatment; tumor imaging examination (breast, chest and abdomen with contrast-enhanced CT or contrast-enhanced MRI, head with MRI, bone scan), 12-lead ECG, echocardiography within 28 days prior to initiation of study treatment. For laboratory tests, imaging evaluation (bone scan allows examination results 2 months before signing the informed consent form), 12-lead ECG, and cardiac colour Doppler ultrasound, if the results were obtained within 14 days before signing the informed consent form, and are sufficient to support the investigator to evaluate the subjects' eligibility, there is no need to do the related examination again during the screening period, otherwise, the examination needs to be repeated during the screening period.

1. Demographics: Including initials, gender, ethnicity, marital status, date of birth, height, and weight;

2. Medical and treatment history: Including past case history and treatment history (clinical/pathological diagnosis, diagnosis time, clinical/pathological stage, HER2/estrogen receptor (ER)/progesterone receptor (PgR) expression; history of surgery, neoadjuvant therapy, adjuvant therapy, radiotherapy; time to progression and diagnosis basis; names, medication regimens, the effectiveness of each line of drugs used in advanced cancer systemic treatment, whether to conduct other therapy, including surgery.), history of other tumors other than breast cancer, smoking and drinking history (frequency, amount, duration), history of drug allergy (name of the drug, allergy symptoms), anamnesis or accompanying diseases/symptoms (name of disease/symptom, name of the combined medication, medication plan, outcome), and bowel habits (frequency);

3. Concomitant medications/treatments: Record the concomitant medication from the screening period to the end of the study treatment, which should include the name of the drug, dose, the route of administration, the frequency of administration, the purpose of administration, and the start and end dates. If the subject starts new systemic anti-tumor therapy during the safety follow-up period, only the concomitant/concomitant therapy for adverse events related to the study drug will be recorded;

4. Blood routine: Including absolute counts of white blood cell, red blood cell, haemoglobin, platelet, neutrophil, and lymphocyte; will be performed on Day 7, 14 and 28 of the first cycle, on Day 28 of subsequent cycles, and at the end/withdrawal of the study if not done within the previous 7 days. If necessary, the investigator can increase the frequency of examinations according to the clinical conditions of the subjects;

5. Urine routine: Including urine protein, urine glucose, urine red blood cells, and urine white blood cells. When urine protein test shows ++ or above, perform a 24-hour urine protein quantification test. Urine routine test will be performed at the end of every 4 cycles and the end/withdrawal of the study (if not performed within the previous 7 days);

6. Stool routine: Including fecal occult blood test, will be performed at the end of every 4 cycles and the end/withdrawal of the study (if not performed within the previous 7 days);

7. Blood biochemical test: including total bilirubin, direct bilirubin, indirect bilirubin, alanine aminotransaminase (ALT), aspartate aminotransferase (AST), alkaline phosphatase (AKP), γ-glutamyl transpeptidase (GGT), lactate dehydrogenase (LDH), total protein, albumin, urea or blood urea nitrogen, serum creatinine, uric acid, fasting blood glucose, triglyceride, cholesterol, potassium, sodium, chloride, calcium, phosphorus, magnesium; and myocardial enzymes if necessary, including but not limited to creatine kinase (CK), creatine kinase isoenzyme (CK -MB), alpha hydroxybutyrate dehydrogenase (butyrate dehydrogenase) enzyme, troponin T (cTnT), as determined by the investigator on a subject basis. Blood biochemical test will be performed on Day 14 and Day 28 of the first cycle, on Day 28 of the subsequent cycles and at the end/withdrawal of the study (if not performed within the previous 7 days);

8. Pregnancy test: Female subjects of childbearing age should undergo blood HCG testing during the screening period and at the end/withdrawal of treatment (if not performed within the previous 7 days);

9. Infectious disease screening: Including the test of five indexes of hepatitis B, HIV antibody, and HCV antibody; will be performed during the screening period. If the hepatitis B and C test results are abnormal, the investigator should determine whether to perform the virus replication (HBV-DNA, HCV-RNA) test.

10. 12-Lead ECG: Will be performed on Day 14 and Day 28 of the first cycle, on Day 28 of the subsequent cycles and at the end/withdrawal of the study (if not performed within the previous 7 days). If the QTc interval increases by >30 msec from the baseline, or if the absolute value of the QTc interval is ≥470 msec in any of the specified ECG measurements, 2 additional ECG examinations (at least 10 minutes apart) are required;

11. Echocardiography: Will be performed at the end of every 4 cycles and the end/withdrawal of the study (if not performed within the previous 7 days). Focus on the change of LVEF, when LVEF decreases to ＜50% and decreases by ≥10% from baseline, or the subject experiences symptoms such as chest pain and palpitations, further examination is required;

12. Vital signs: Including body temperature, blood pressure, respiratory rate, and heart rate; will be performed on Day 14 and Day 28 of the first cycle, on Day 28 of the subsequent cycles and at the end/withdrawal of the study (if not performed within the previous 7 days);

13. Physical examination: Including evaluation of the head, skin, lymph nodes, eyes, ears, nose, throat, oral cavity, respiratory system, cardiovascular system, abdomen, genitourinary system, musculoskeletal system, nervous system and mental state; will be performed at the end of every 4 cycles and the end/withdrawal of the study (if not performed within the previous 7 days). Comprehensive physical examination results during the screening period and at the end of the study will be recorded, and for physical examinations during the study treatment, only abnormalities will be recorded;

14. Adverse events: Will be monitored from informed consent to at least 28 days after the last dose. The adverse events, concomitant medications/treatments and unscheduled examinations during the period were recorded in detail, until the adverse events were relieved, or the subjects started new antitumor treatment or lost to follow-up, or reached a stable level;

15. Tumor imaging evaluation: Imaging evaluation in the screening period will focus on breast, chest and abdomen, and craniocerebral, neck, and pelvic when clinical indications show it is necessary. Enhanced CT, PET-CT or MRI can be used, and enhanced MRI is the first choice for intracranial lesion examination. The baseline tumor assessment can be performed within 4 weeks before the first dose, and CT/MRI scan results obtained before signing the informed consent can be used as long as they conform with all other requirements of this protocol. Routine bone scans are generally not required in patients unless bone metastases are suspected. Investigators may add scan sites to baseline or subsequent tumor assessments as clinically warranted.

Imaging examinations during the trial period should be performed under the same conditions as the baseline examinations (contrast agent use, etc.), every 2 cycles for the first 18 cycles, and every 3 cycles thereafter. Bone scans are performed when bone progression is suspected or when CR confirmation is performed. If new lesions are suspected, imaging examinations could be performed in time. The first PR/CR should be confirmed after 4 weeks. The time window allowed for tumor imaging was ±7 days, with unscheduled imaging when disease progression (eg, worsening symptoms) was suspected.

16. Disease progression: In subjects who exit the trail for reasons beyond disease progression confirmed by imaging and have not been evaluated by imaging within 4 weeks before, imaging evaluation should be performed at the end of the treatment. Meanwhile, follow-up of tumor response should be continued at the protocol-specified follow-up frequency after the end of the trial until documented disease progression or the initiation of new tumor therapy.

17. Death: After the trial treatment was discontinued, the survival status and subsequent anti-tumor treatment could be collected through clinical follow-up or telephone follow-up every 3 months until death.

18. Pyrotinib: once a day, 400 mg each time, orally administered within 30 minutes after breakfast, continuous administration for 28 days as a cycle.

19. SHR6390: once a day, 125mg each time, orally administered for 3 weeks followed by 1 week off, 4 weeks as a cycle. It is recommended to be taken at the same time of day, with warm water, and fasting for at least 1 hour before and after taking the medicine.

The above medication can be adjusted based on the adverse reactions of the subjects according to the protocol. Subjects will continue medication until disease progression, unacceptable toxicity, withdrawal, or discontinuation of medication at the discretion of the investigator.

20. Drug distribution/recovery: Except for the first cycle where only drugs are distributed and the end of treatment where only drugs are recovered, the drugs are recovered and distributed at the end of each cycle, and corresponding records are made.

21. Subsequent anti-tumor therapy: It is necessary to record whether subjects were treated with other regimens during the period from exit to the end of survival follow-up; there is no need to record co-medication for other diseases.

22. Safety follow-up: Within at least 28 days after the last study medication, the investigator can select blood routine, blood biochemistry, electrocardiogram, echocardiography, physical examination, vital signs, ECOG score and other clinical evaluation measures according to the specific conditions of the subjects, and record as unscheduled examination. Drug-related adverse events should be followed up until disappearance, remission to baseline level or ≤ grade 1, stable status, or reasonable explanation (whichever comes first);

23. Efficacy follow-up: Subjects exit for reasons except PD and death need to be followed up for efficacy. Tumor imaging evaluation should be performed at least once every 12 weeks (±7 days) from the last tumor imaging evaluation during the study period, until disease progression, initiation of other anti-tumor drugs or death (whichever comes first), and detailed records of the date and tumor imaging evaluation results of each follow-up should be made;

24. Survival follow-up: All non-dead subjects who complete safety follow-up and efficacy follow-up (whichever completed later) will be required to undergo survival follow-up. From the date of completing the safety follow-up and efficacy follow-up (whichever completed later), survival information (date of death and cause of death) will be collected every 12 weeks (± 7 days) at least once every 12 weeks (± 7 days) by telephone inquiries or clinical follow-up of the subjects, their family members or local physicians until death, loss to follow-up, or end of OS data collection, whichever occurs first. The details of each survival follow-up should be recorded and entered in the corresponding eCRF form.

Abbreviations

Abbreviation/Term	Defination
ADL	Activities of daily living
AE	Adverse event
AKP/ALP	Alkaline phosphatase
ALB	Albumin
ANC	Absolute neutrophil count
AST	Aspartate aminotransferase
BUN	Blood urea nitrogen
NMPA	National Medical Products Administration
CBR	Clinical benefit rate
CD-ROM	Compact Disc Read-Only Memory
CR	Complete response
ctDNA	Circulating tumor DNA
CHOL	Cholestenone
CRA	Clinical research associate
CRC	Clinical research coordinator
CRF	Case report form
CT	Computed tomography
Cr	Creatinine
CNS	Central Nervous System
CSF	Cerebrospinal fluid
DOR	Duration of response
DCR	Disease control rate
ECOG	Eastern Cooperative Oncology Group
EC	Ethic committee
EDC	Electronic data collection
EGFR	Epidermal growth factor receptor
ER	Estrogen receptor
FAS	Full analysis set
FISH	Fluorescence in situ hybridization
FGF	Fibroblast growth factor
Hb	Hemoglobin
HCV	Hepatitis C virus
ITT	Intend to treat
LVEF	Left ventricular ejection fraction

PPS	Per-protocol set
PI	Priniciple invertigator
SS	Safety set
GCP	Good clinical practice
HFS	Hand foot syndrome
ICF	Informed consent form
mPFS	Median progression-free survival
MRI	Magnetic resonance imaging
NCI-CTC	National cancer institute common terminology criteria
NE	Not evaluated
ORR	Objective response rate
OS	Overall survival
PD	Progressive disease
PFS	Progression-free survival
PLT	Platelet
PR	Partial response
PR	Progesterone receptor
RECIST	Response Evaluation Criteria In Solid Tumors
RBC	Red blood cell count
RTK	Receptor tyrosine kinase
SAE	Serious adverse event
SD	Stable disease
SRT	Stereotactic radiotherapy
ULN	Upper normal limit
VEGF	Vascular endothelial growth factor
WBC	White blood cell count
WBRT	Whole brain radiotherapy

1 Research Background

Redacted

Redacted

Redacted

Redacted

Redacted

2 Study Objective and Endpoints

2.1 Study Objective

To evaluate the efficacy and safety of pyrotinib combined with CDK4/6 inhibitor SHR6390 in the treatment of HER2-positive (HR-positive and negative) metastatic breast cancer.

2.2 Endpoints

2.2.1 Primary endpoint

Objective response rate (ORR) by investigators according to RECIST 1.1 criteria: the proportion of subjects with CR and PR.

2.2.2 Secondary endpoints

Progression free survival (PFS) and overall survival (OS) by investigators according to RECIST 1.1 criteria:

- PFS: defined as the time from the start of the subject receiving study treatment to the first imaging confirmed disease progression (PD) or death from any reason;
- OS: Refers to the time period from the date of receiving study treatment to the date of death (any cause).

Safety measurements: ECOG PS, vital signs, physical examination, laboratory indicators (blood and urine routine urine, stool test, and blood biochemistry test), ECG, echocardiography, adverse events (AE), and serious adverse events (SAE), as assessed by NCI-CTCAE 5.0.

3 Study Design

This is a single-arm, open-label, single-center, phase II clinical study. According to the "Administrative Measures for Drug Registration", "Quality Management Practice for Drug Clinical Trials", and "Guidelines for Clinical Pharmacokinetic Trials of New Drugs (Chemical Drugs)", this trial intends to evaluate the efficacy and safety of pyrotinib combined with CDK4/6 inhibitor SHR6390 and explore a reasonable dose of the dual combination in patients with HER2+ advanced breast cancer.

After screening, eligible subjects will enter the trial period and received treatment with pyrotinib and SHR6390 until the disease progressed, or intolerable toxicity, or the informed consent withdrawn, or the investigator judged that the drug must be discontinued. The imaging evaluation during the study will be performed according to RECIST 1.1 criteria, and the evaluation result of the investigator site will be the final result.

Based on Simon's two-stage design, 23 subjects will be enrolled in In the first stage, if ≤12 achieve CR/PR, it will be deemed invalid and the study will be terminated, otherwise the study will go to the second stage, and continue to enroll total of 37 patients. If more than 23 patients achieve

CR/PR, the study treatment will be considered to be effective. Considering a dropout rate of 10%, totally no more than 41 patients would be enrolled.

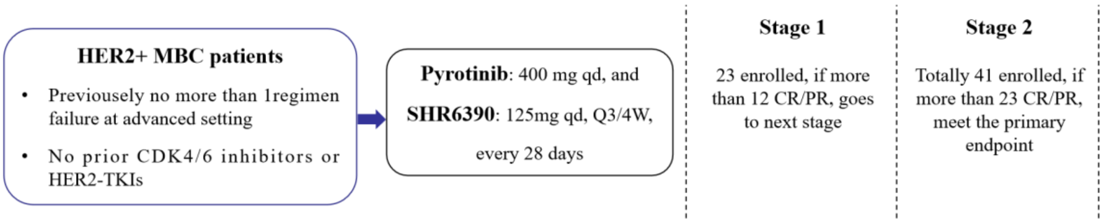

During the study, ORR, PFS, OS and safety will be evaluated and statistically described according to the prespecified criteria of the protocol.

4 Selection and Withdrawal of Patients

4.1 Inclusion Criteria

Patients must meet all of the following inclusion criteria to be enrolled in this trial:

1. Patients with HER2-positive advanced breast cancer who had received ≤ 1-line treatment in the past;
2. Female ≥ 18 years and ≤ 70 years of age;
3. HER2-positive breast cancer is defined as immunohistochemistry (IHC) score of 3+, or IHC score 2+ and positive in situ hybridization (ISH) test (ISH amplification rate ≥2.0) confirmed by the pathology laboratory according to 2018 ASCO-CAP guidelines;
4. Eastern Cooperative Oncology Group(ECOG) performance status 0 to 1;
5. Life expectancy is not less than 12 weeks;
6. At least one measurable lesion according to RECIST 1.1;
7. Patients must have adequate organ function, criteria as follows:

1) Blood routine test:
   Absolute neutrophil count (ANC) ≥ 1.0×10^9/L (without growth factor within 14 days);
   Platelet count (PLT) ≥ 100×10^9/L (without corrective treatment within 7 days);
   Haemoglobin (Hb) ≥ 90 g/L (without corrective treatment within 7 days);
2) Blood chemistry test:
   Total bilirubin (TBIL) ≤ 1.5 times the upper limit of normal (ULN);
   Alanine aminotransaminase (ALT) and aspartate aminotransferase (AST) ≤ 3 times ULN; for patients with liver metastases, ALT and AST ≤ 5 times ULN;
   Blood urea nitrogen (BUN) and serum creatinine (Cr) ≤ 1.5 times ULN;
3) Echocardiography:
   Left ventricular ejection fraction (LVEF) ≥ 50%;
4) 12-lead ECG
   The QT interval corrected by Fridericia's formula (QTcF) is < 470 ms for females.

8. Patients need to voluntarily join this study after they fully understand and sign the informed consent form. Patients need to have good compliance and be willing to cooperate with follow-up.

4.2 Exclusion criteria

Patients with any of the following conditions will not be selected as a subject:

1. Clinically symptomatic patients with brain metastases
2. Patients with dysphagia, chronic diarrhoea and intestinal obstruction and factors that affect the administration and absorption of the drug;
3. Patients who have received radiotherapy, chemotherapy, surgery(excluding local puncture) within 4weeks before enrolment; patients who have received anti-tumor endocrine therapy during screening;
4. Patients who participate in clinical trials of other new drugs within 4 weeks before enrolment;
5. Concurrently treated, or previously treated with HER2 tyrosine kinase inhibitors (including lapatinib, neratinib, pyrotinib, etc.);
6. Prior any CDK4/6 inhibitors;
8. History of other malignant tumours within 5 years, excluding cured cervicalcarcinoma in situ, skin basal cell carcinoma or skin squamous cell carcinoma;
9. Female patients during pregnancy and lactation; fertile female patients who testedpositive on a baseline pregnancy test; female patients of childbearing age who areunwilling to take effective contraceptive measures during the trialor within 90 days after last dose of study drug;
10. Allergic to the study drug; history of immunodeficiency,including HIV positive, HCV or other acquired or congenital immunodeficiency diseases, or history of organ transplantation;
11. Severe heart disease, including but not limited to the following:
    - History of heart failure or systolic dysfunction (LVEF < 50%);
    - Angina or arrhythmia of high risk or requiring treatment (eg, second-degree type 2 AV block or third-degree AV block, ventricular tachycardia);
    - Clinically significant heart valve disease;
    - Transmural myocardial infarction shown by ECG;
12. According to the judgment of the investigator, there are concomitant diseases that seriously endanger the patient's safety or affect the completion of the study (including but not limited to severe hypertension that cannot be controlled by drugs, severe diabetes, active infection, etc);
13. Complicated with moderate infection within 4 weeks before the first dose (eg: intravenous infusion of antibiotics, antifungal or antiviral drugs is required according to clinical diagnosis and treatment standards), or unexplained fever >38.5°C during screening/before the first dose;
14. Any other circumstances that are not suitable for inclusion in this studyjudged by investigator.

4.3 Treatment discontinuation Criteria

4.3.1 Treatment discontinuation Criteria

Discontinuation study follow-up:

1. Any time when subjects withdraw their informed consent and request to withdraw;

Discontinuation study treatment but follow-up according to protocol:

1. Imaging or clinical evidence progress, unless the subject can benefit from continued treatmentjudged by the investigator (participants with BM at baseline getting isolated intracranial progression could continue study after brain radiotherapy till second

progression if they would benefit from the treatment according to the physician discretion);

2. Any clinical adverse events, abnormal laboratory tests or other medical conditions that may preventthe subjects from benefiting from continued medication;
3. Those who seriously violated the trial protocol and the investigators assessed that the treatment should be terminated;
4. During the research process, the subject has a pregnancy event;
5. Other reasons considered by the investigator to be unable to continue the study drug treatment.

4.3.2 Treatment of withdrawn subjects

Be sure to make every effort to complete the end/withdrawal visit according to the protocol. For subjects who terminate the study treatment, the safety follow-up, efficacy follow-up (if necessary) and survival follow-up must be carried out according to the protocol.

Investigators could suggest or provide new or alternative treatments to patients based on their actual conditions.

4.4 Criteria for exclusion from analysis

1. Does not meet the inclusion criteria, or meets the exclusion criteria;
2. Incomplete data affect the judgment of efficacy and safety;
3. The dosage, method and course of drug use do not conform to the plan, affecting the evaluation of efficacy;
4. Use prohibited drugs specified in the protocol.

4.5 Trail termination criteria

The study may be terminated or suspended with good reason. If the study is terminated or suspended, the investigator site (Henan Cancer Hospital) will submit a written notice stating the reasons for early termination or suspension to the relevant departments. The principal investigator must immediately report to the ethics committee and provide appropriate reasons.

Termination criteria include but are not limited to the following

1) Unexpected, significant or unacceptable risks to the subject are found;
2) Major mistakes were found in the plan during the execution of the test;
3) The study drug/treatment is ineffective, or it is meaningless to continue the trial;
4) Completion of the trial is extremely difficult due to reasons such as severe delays in subject enrollmentor frequent protocol deviations.

4.6 Lifestyle requirements

4.6.1 Contraception

Women of childbearing age must undergo a serum pregnancy test prior to enrollment and must be negative for enrollment.

Female subjects of childbearing age should be sterilized or must agree to use a highly effective method of contraception during the study and within 90 days after the last dose of study drug.

After consultation with the subject, the investigator or an authorized designated person shall select the contraceptive measures suitable for her and her partner from the following contraceptive methods, and shall confirm that the subject knows how to use the contraceptive method correctly and consistently. At the time points of study visits listed in the schedule, the investigator would inform the subject the need for continuous and correct contraception. In addition, subjects are advised to notify the investigator immediately if they discontinue the chosen method of contraception, or have a suspected or confirmed pregnancy.

High-efficiency contraceptive methods are those that are used consistently and correctly, alone or in combination with other methods, with a failure rate of less than 1% per year. Including the following:

1. commonly used hormonal contraceptive methods (eg, oral, insertion, injection, implant, transdermal) associated with suppression of ovulation, provided that: the female subject has been using the method for a period of time, and proven effective, it is planned to continue to be used correctly throughout the study period.
2. The IUD is correctly placed.
3. Concomitant use of topical spermicide (ie foam, gel, film, cream or suppository) with male/female condoms.
4. Males are sterilized by vasectomy.
5. Bilateral tubal ligation/bilateral salpingectomy or bilateral tubal occlusion surgery (occlusion surgery has been proven effective by relevant instruments).

5 Study treatment

5.1 Overview of study drugs

5.1.1 Drugs and provider

The study drugs SHR6390 and pyrotinib are produced and provided by Jiangsu Hengrui Pharmaceutical Co., Ltd.

5.1.2 Dosage form and strength

1) SHR6390: 25 mg or 50mg tablets

2) Pyrotinib: 80 mg or 160mg tablets

5.1.3 Storage requirements

- SHR6390: Store sealed at temperatures below 25 ℃. It is valid for 24 months tentatively.
- Pyrotinib: Store at temperatures below 25 ℃ in dry place. It is valid for 12 months.

5.2 Dosage regimen

- Pyrotinib: once a day, 400 mg each time, orally administered within 30 minutes after breakfast, continuous administration for 28 days as a cycle.
- SHR6390: once a day, 125 mg each time, orally administered for 3 weeks on and 1 week off, and 4 weeks as a cycle. It is recommended to take the medicine at about the same time every day, with warm water, and fast for at least 1 hour before and after taking the medicine.

The above medication can be adjusted based on the adverse reactions of the subjects according to the protocol. Subjects will continue medication until disease progression, unacceptable toxicity, withdrawal, or discontinuation of medication at the discretion of the investigator. The medication cycle will be determined since the date of the first dose. If any test drug is suspended, missed, or underused during the study, the drug will continue to be administered according to the plan cycle, and no supplement or cycle adjustment will be given. However, it should be recorded in the original data in detail. If there is a miss, the time when the missed drug should have been used and the reason for the missed use should be recorded in detail; if there is a less use due to various reasons such as adverse drug reactions, it should be recorded in the patient diary, the original medical record and the eCRF.

5.3 Dosage adjustment

If there was a toxic reaction, investigators could give corresponding treatment according to the situation. The suggested treatment principles are as follows:

When there is a toxicity clearly related to the study drug, the investigator will deal with it according to clinical manifestations. Study drugs could be suspended till the toxicity recovering to ≤ grade 1 (or recovering to ≤ grade 2 and tolerable to the subjects and have no obvious safety risk) judged by the investigator. If the adverse event recurs, the investigator would interrupt or decrease the dose according to the clinical situation, and protect subjects' safety to the greatest extent. If the toxicity cannot be recovered within 3 weeks after drug suspension, the subjects should withdraw from the study in principle. The duration of suspension should be included in the dosing cycle.

The initial dose of pyrotinib and SHR6390 may be adjusted according to the subjects' tolerance. It is planned that after 6 patients enrolled, the investigators will discuss whether it is necessary to reduce the dose and adjust the study protocol.

Table 5 Recommended Dosage Adjustment for Adverse Event

Adverse events NCI CTCAE 5.0	Grade			
	1	2	3	4
Hematological toxicity	Maintain the dose	Maintain the dose	Suspend treatment and symptomatic treatment till be recovered to ≤ grade 1. According to the investigator's judgment, the dose can be maintained or reduced in this cycle and subsequent cycles.	Suspend treatment and symptomatic treatment till be recovered to ≤ grade 1. According to the investigator's judgment, the dose will be reduced in this cycle and subsequent cycles.
Non-hematological toxicity	Maintain the dose	Maintain the dose or suspend treatment and symptomatic treatment, till be recovered to ≤ grade 1. According to the investigator's judgment, the dose can be maintained or reduced in this cycle and subsequent cycles.	Suspend treatment and symptomatic treatment till be recovered to ≤ grade 1. According to the investigator's judgment, the dose will be reduced in this cycle and subsequent cycles.	Permanently discontinue medication, withdraw from study

Febrile neutropenia	-	-	Suspend treatment and symptomatic treatment till be recovered to ≤ grade 1. According to the investigator's judgment, the dose will be reduced in this cycle and subsequent cycles.	Permanently discontinue medication, withdraw from study

During the trial, multiple suspensions or multiple adjustments of the dose are allowed. The dose of pyrotinib will be adjusted according to the gradient of 400 mg, 320 mg and 240 mg; the dose of SHR6390 will be adjusted according to the gradient of 125 mg, 100 mg and 75 mg. If there are still intolerable or uncorrectable adverse events after adjusting to the lowest dose level, the drug should be stopped.

5.4 Common adverse reactions and its treatment

In the event of adverse events during the study, investigators should take active symptomatic treatment, and make detailed records of concomitant treatment in progress notes and EDC system. Investigators should carry out medical treatment according to the actual clinical situation. The following treatment methods are for reference:

1) Diarrhea: Before subjects start the study treatment, the investigator should inform in detail the possibility of diarrhea and the treatment measures for diarrhea and prophylactic dispensing loperamide and montmorillonite powder. When diarrhea occurs, symptomatic treatment should be given first, followed by close follow-up or observation (≤14 days). It is recommended to start loperamide immediately when diarrhea occurs (4 mg for the first time, and subsequently 2 mg at each time the stool is not formed, up to 16 mg/day). If the diarrhea does not improve, montmorillonide powder would be added as 3 times/day and 3 g each time. These treatment will be continued until the diarrhea stops more than 12 hours. For severe diarrhea, oral or intravenous electrolytes can be administered. For grade 3 diarrhea that cannot be relieved though treating as above or grade 1-2 diarrhea with complications, it is recommended to suspend pyrotinib until diarrhea recovered to grade 1.

2) Hand-foot syndrome and rash: symptomatic treatment would be given first and follow up closely. Suggested symptomatic and supportive care include intensive skin care, keeping skin clean, avoiding secondary infection, avoiding pressure or friction, using moisturizer or lubricant, topical lotion or lubricant containing urea and corticosteroids, and topical antifungal or antibiotic treatment as necessary. For the event of hand-foot syndrome cannot be recovering though treating as above, it is recommended to suspend pyrotinib until diarrhea reliving to grade 1.

3) Abnormal liver function: investigators shall give symptomatic treatment for protecting liver function or observation according to the conditions of subjects and AEs, and the frequency of blood biochemical test shall be increased according to clinical needs. The study treatment would be

suspended or reduced if there was still abnormal liver function of grade 2 or above after 14 days of active treatment or observation. Dose interruption would be continued until the AE recovering to grade 1. Dose reduction is allowed if necessary. For subjects with liver metastasis, if ALT/AST exceeds 1.5 × UNL at baseline, liver function should be closely monitored to determine whether the subjects are suitable for inclusion in this study.

4) Vomiting: symptomatic treatment first and follow up closely. For the vomiting of grade 2 or above cannot be recovering though treating as above, it is recommended to suspend or reduce the dosage of study drugs until the AE recovering to ≤ grade 1. If the time vomiting happened closely to the time of taking the medicine, it is necessary to record the occurrence time of the vomiting in detail. However, regardless of whether the vomiting affects the absorption of the study drug, the drug will continue to be taken according to the scheduled cycle, without supplementary or cycle adjustment.

5) Neutropenia: blood routine test is required before subjects start the study treatment. If the result showed ANC < 1000/mm^3, or platelets < 50,000/ mm^3, SHR6390 would not be recommended. Do not suspend SHR6390 unless grade 3 neutropenia or febrile neutropenia occurs, if grade 3 neutropenia persisted for > 1 week after suspending or grade 3 neutropenia recurred on day 1 of subsequent cycles, a dose reduction of SHR6390 is recommended when restarting treatment determined by the investigator based on the actual clinical situation.

Table 6 Recommended Pyrotinib Dosage Adjustment

NCI-CTC AE 5.0*	Dose suspension	Dose adjustment after the suspension
**Cardiotoxicity**		
Clinically significant grade ≥ 2 LVEF decline/LVEF below the lower limit of normal (including asymptomatic decline in LVEF ≥ 10% and LVEF < 50%, or heart failure)	Permanently discontinued	-
**Diarrhea**		
Grade 4	Permanently discontinued	-
Grade 3	Suspended until recovering to grade ≤ 1 and complication resolving	First adjustment: 400 mg;<br>Second adjustment: 320 mg
Grade 1-2 with complications (including but not limited to grade ≥ 2 nausea or vomiting, decreased ECOG PS, fever, sepsis, neutropenia, bleeding, and dehydration)		
**Other AEs**		
Nonhematologic AEs of grade ≥ 2 (excluding alopecia, fatigue, asthenia, etc.)	Suspended until recovering to grade ≤ 1	First adjustment: 400 mg;<br>Second adjustment: 320 mg
Hematologic AEs of grade ≥ 3	Suspended until recovering to grade ≤ 1	First adjustment: 400 mg;<br>Second adjustment: 320 mg

Table 7 Recommended SHR6390 Dosage Adjustment for hematologic AEs

NCI-CTC AE 5.0	Dose adjustment
Grade 1 or 2	No need to dose adjustment

Grade 3	Suspended, and retest complete blood cell count within 1 week. Restarted when AEs recovering to grade 2 or below, and resuming with the original dose<br>If Grade 3 neutropenia persists for > 1 week, reduce dose when resuming
Grade 3 with fever (≥38.5 ℃) and/or infection	Suspended until recovering to ≤ Grade 2, and reduce dose when resuming
Grade 4	In any circumstances:<br>Suspended until recovery to ≤ Grade 2 and consider G-CSF<br>Reduce dose when resuming

5.5 Investigational products management, distribution and recycle

The management, distribution, and recycle of clinical drugs in this trial are handled by dedicated personnel. Investigators must ensure that all investigational products are used only for subjects participating in clinical trials, and their dosage and usage should follow the trial protocol, and the remaining drugs should be returned to sites. Do not transfer clinical drugs to any non-clinical trial participants.

Receipt must be signed when experimental drug is distributed. The remaining medicines and empty boxes are recovered after the study. The distribution and recycle of each medicine should be recorded in a timely manner on a special record sheet.

The monitor is responsible for supervising the supply, use, storage and disposal of surplus drugs in clinical trials.

5.6 Patient compliance

If test drugs have been assigned to individual subjects, these subjects should be required to return unused test drugs each time they return during follow-up. The subjects recorded the drug information on the diary card every day.

When the subject is taking medication, the sites will evaluate the compliance of the investigational products at each treatment visit (except follow-up visit). Record the number of returned investigational products, compare the returned investigational products with the dose information reported by the subject, and compare with the prescribed dose to monitor compliance. Compliance and reasons for deviation will be recorded in source files and drug inventory records. Any deviation from compliance should be explained accordingly.

Calculation of drug dose compliance: compliance (%) = number of tablets taken/ expected number of tablets to be taken×100.

If the compliance percentage calculated according to the above formula is less than 80% or greater than 120%, it can be considered that the subject does not comply with the dose. If the calculated percentage exceeds the above range, it shall be recorded as a protocol deviation. The eCRF of the investigational product should reflect the verified drug dosage information provided by the subject.

6 Concomitant Therapy

Concomitant medications include all medications (eg, prescription, over-the-counter, natural or homeopathic, nutritional supplements) the subject used from screening to treatment. All concomitant medications should be reported to the investigator and recorded on the concomitant medication electronic case report form (eCRF). Concomitant medication and treatment information should include date, reason, drug prescription, treatment or method, and any relevant clinical results, with particular attention to drugs associated with adverse events associated with the investigational drug.

6.1 Prohibited Drugs during the study

1) During the treatment period, other anti-tumor drugs and complementary drugs related to tumor treatment other than the study drugs specified in this protocol should be stopped, including anti-tumor Chinese medicines and immunologic agents;
2) Systemic high-dose corticosteroids, refers to the daily dose of dexamethasone > 20 mg (or equivalent dose of other corticosteroids), for > 7 days;
3) Any other experimental drug except the experimental drug used in this study;
4) Products containing St. John's wort (Hypericum perforatum) are prohibited.

6.2 Drugs to be used with caution during the study

If the subjects have adverse reactions, they should be closely observed, and if necessary, actively treat the symptoms, and record and explain the drugs used on the eCRF form. The following drugs should be used with caution during the research:

1) Drugs and foods that interfere with Cytochrome P450 proteins, including:

- CYP3A4 inducers (Dexamethasone, phenytoin sodium, carbamazepine, rifampicin, rifabutin, rifapentin) and inhibitors (ketoconazole, itraconazole, erythromycin, clarithromycin, atazanavir, indinavir, nefazodone, nelfinavir, ritonavir, saquinavir, telithromycin, voriconazole, citrus paradisi macf., etc.);
- Substrates of CYP3A4 (Simvastatin, pimozide);
- Other drugs metabolized by CYP3A4 (benzodiazepines, dihydropyridine, calcium ion antagonist HMG-COA reductase inhibitor);
- Substrates of CYP2C9 (Diclofenac, phenytoin, piroxicam, S-warfarin and tolbutamide) and substrates of CYP2C19 (Diazepam, Imipramine, Lansoprazole and S-Mephenytoin).

2) Drugs that prolong the QT interval: including antibiotics, antiarrhythmic, antipsychotic, antifungal, antimalarial and antidepressant drugs (such as clarithromycin, quinidine, risperidone, fluconazole, mefloquine, amitriptyline, azithromycin, sotalol, fluphenazine, ketoconazole, chloroquine, imipramine, erythromycin, amiodarone, droperidol, clomipramine, roxithromycin, disopyramide, haloperidol, Dosulepin, metronidazole, procainamide, thioridazine, doxepin, moxifloxacin, pimozide, olanzapine and clozapine).

6.3 Drugs and treatments that can be used in combination during the study

Patients can receive the best supportive treatment, and actively treat the clinical complications and various AEs. Patients with bone metastases who have started bisphosphonate treatment before enrolment can continue to use it during the trial; during the study period, the investigator used bisphosphonate therapy as appropriate according to the patient's condition. In strict accordance with the GCP guidelines, all drugs used in combination are recorded in the eCRF. From the 4 weeks before the study treatment to the end of the safety follow-up, the combination medication/treatment should be recorded.

G-CSF (granulocyte colony-stimulating factor) is not permitted as primary prevention, but it can be used to treat neutropenia that occurs during treatment according to the current guidelines by American Society of Clinical Oncology.

Erythropoietin may be used in the supportive treatment of anemia at the discretion of the investigator.

6.4 Dietary requirements

Grapefruit, black mulberry, wild grape, pomegranate, black raspberry, and beverages and foods containing the above fruit ingredients can interfere with liver P450 enzymes, and it is not recommended to consume with study drugs.

7 Study Procedures

Before starting the study, patients must read and sign the current ethics committee (EC) approved informed consent form. All study steps must be performed within the time frame specified in the study plan.

7.1 Screening

After signing the informed consent form, the subjects entered the screening period. Physical examination, vital signs and laboratory tests (including blood routine, urine routine, liver and kidney function, blood lipids, blood electrolytes, fasting blood glucose, blood HCG, etc.) are conducted within 14 days before the start of study treatment; tumor imaging examination (breast, chest and abdomen with contrast-enhanced CT or contrast-enhanced MRI, head with MRI, bone scan), 12-lead ECG, echocardiography within 28 days prior to initiation of study treatment. For laboratory test, imaging evaluation (bone scan allows examination results 2 months before signing the informed consent form), 12-lead ECG, and cardiac colour Doppler ultrasound, if the results were obtained within 14 days before signing the informed consent form, and are sufficient to support the investigator to evaluate the subjects' eligibility, there is no need to do the related examination again during the screening period, otherwise, the examination needs to be repeated during the screening period. All the examinations below must be completed within 28 days prior to the trial period.

- Demographics: Initials, gender, race, marital status, date of birth, height, weight;
- Medical history: Past medical history and treatment history of breast cancer (clinical/pathological diagnosis, diagnosis time, clinical/pathological stage, HER2/ER/PR

expression, whether to surgery, neoadjuvant therapy, adjuvant therapy, radiotherapy, and when to progress, diagnosis progress basis, medications previously used, usage, duration, efficacy, and whether to conduct other therapy, including surgery.), history of other tumors other than breast cancer, smoking and drinking history (frequency, amount, duration), history of drug allergy (name of drug, allergy symptoms), anamnesis or accompanying diseases/symptoms (name of disease/symptom, name of combined medication, medication plan, outcome), and bowel habits (frequency);

- ECOG PS;
- Vital Signs: Including body temperature, blood pressure, respiratory rate, and heart rate. Smoking and coffee are banned for 30 minutes before a blood pressure measurement, and subject would take a quiet rest for at least 10 minutes, and then sit in a seated position and place their elbow at heart level during the measurement (record as left or right);
- Physical examination: Including evaluation of head, skin, lymph nodes, eyes, ears, nose, throat, oral cavity, respiratory system, cardiovascular system, abdomen, genitourinary system, musculoskeletal system, nervous system and mental state;
- Blood routine: Including absolute counts of white blood cell, red blood cell, haemoglobin, platelet, neutrophil, lymphocyte;
- Urine routine: Including urine protein, urine glucose, urine red blood cells, and urine white blood cells. When urine protein test shows ++ or above, perform 24-hour urine protein quantification test;
- Stool routine: Including fecal occult blood test;
- Blood biochemical test: including total bilirubin, direct bilirubin, indirect bilirubin, alanine aminotransaminase (ALT), aspartate aminotransferase (AST), alkaline phosphatase (AKP), γ-glutamyl transpeptidase (GGT), lactate dehydrogenase (LDH), total protein, albumin, urea or blood urea nitrogen, serum creatinine, uric acid, fasting blood glucose, triglyceride, cholesterol, potassium, sodium, chloride, calcium, phosphorus, magnesium; and myocardial enzymes if necessary, including but not limited to creatine kinase (CK), creatine kinase isoenzyme (CK - MB), alpha hydroxybutyrate dehydrogenase (butyrate dehydrogenase) enzyme, troponin T (cTnT), as determined by the investigator on a subject basis;
- Infectious disease screening: Including the test of five indexes of hepatitis B, HIV antibody, and HCV antibody; will be performed during the screening period. If the hepatitis B and C test results are abnormal, the investigator should determine whether to perform the virus replication (HBV-DNA, HCV-RNA) test;
- Pregnancy test;
- 12-Lead ECG: If QTc interval increases by >30 msec from the baseline, or if the absolute value of the QTc interval is ≥470 msec in any of the specified ECG measurements, 2 additional ECG examinations (at least 10 minutes apart) are required;

- Echocardiography;
- Tumor imaging evaluation: Imaging evaluation in the screening period will focus on breast, chest and abdome, and craniocerebral, neck, and pelvic when clinical indications show it is necessary. Enhanced CT, PET-CT or MRI can be used, and enhanced MRI is the first choice for intracranial lesion examination. The baseline tumor assessment can be performed within 4 weeks before the first dose, and CT/MRI scan results obtained before signing the informed consent can be used as long as they conform to all other requirements of this protocol;
- Concomitant medications/treatments:Record the concomitant medication from the screening period;
- Adverse events: Will be monitored from informed consent to at least 28 days after last dose.

After completing all of the above screening assessments, eligible persons receive a subject number.

7.2 Trial period

The treatment period begins with the subject's first dose. The first study drug should be administered as closely as possible to the time when the screening assessments are completed and the inclusion and exclusion criteria are confirmed, and 28 days make up a treatment cycle.

Visits will be conducted once on Day 14 (±3), Day 28 (±3) of Cycle 1, and once on Day 28 (±3) of Cycle 2 and thereafter. Investigators can increase the examination items or increase the frequency of visits according to the clinical conditions of the subjects to ensure the safety of the subjects to the greatest extent.

The following examinations should be completed within the time window listed in the flow chart (except for the time window of ±7 days for tumor imaging examinations that is specifically stated, the time window for other items is ±3 days from the first cycle). In case of statutory holidays, the visit can be advanced accordingly, and the reason for exceeding the window shall be recorded in the eCRF. If the subjects are out-of-town patients, the following examinations (***except*** **tumor imaging examinations**) can be completed in local hospitals from the second cycle and the results will be notified to the investigator, who will determine whether it is necessary to return to the research center for further examination.

- ECOG PS, vital signs, blood biochemical test, 12-Lead ECG: On Day 14 and Day 28 of Cycle 1, and on Day 28 of Cycle 2 and thereafter. If QTc interval increases by >30 msec from the baseline, or if the absolute value of the QTc interval is ≥470 msec in any of the specified ECG measurements, 2 additional ECG examinations (at least 10 minutes apart) are required;
- Blood routine will be performed on Day 7, 14 and 28 of the first cycle, on Day 28 of subsequent cycles, and at the end/withdrawal of the study if not done within the previous 7 days. If necessary, the investigator can increase the frequency of examinations according to the clinical conditions of the subjects;
- Physical examination: Will be performed at the end of every 4 cycles;

- Echocardiography: Will be performed at the end of every 4 cycles. Focus on the change of LVEF, when LVEF decrease to ＜50% and decrease by ≥10% from baseline, or the subject experiences symptoms such as chest pain and palpitations, further examination is required;
- Urine/stool routine: Will be performed at the end of every 4 cycles;
- Tumor imaging evaluation: The time points of tumor imaging during dosing were determined from the start of study treatment, regardless of the time during which dosing was suspended due to toxicity. The allowable time window for tumor imaging is ±7 days, and the specific evaluation time points are as follows:
  - ✓ First evaluation will be conducted on Day 28 of Cycle 2, and every 2 cycles for first 18 cycles, and every 3 cycles thereafter untildisease progression, intolerable toxicity, or initiation of new tumor therapy;
  - ✓ The first PR/CR should be confirmed after 4 weeks, and the evaluation for confirming would not change the time points fixed before;
  - ✓ Perform tumor assessment at the end of treatment or subject withdrawal (if not done within the previous 4 weeks).

  Note: Imaging examinations during the trial period should be performed under the same conditions as the baseline examinations (contrast agent use, etc.), every 2 cycles for first 18 cycles, and every 3 cycles thereafter. The chest, abdominal lesions, and bone lesions (if any) confirmed at baseline should be examined; other lesions found at baseline except for the above, or suspected new lesions at later stage should also be examined in a timely manner. Unscheduled imaging studies may be performed when disease progression is suspected (eg, worsening symptoms) or when a PR or CR evaluation is required (4 weeks after the evaluation). For patients with bone metastases at baseline, bone scans should be performed at least once every 8 cycles (about 8 months). Generally, patients do not need routine bone scans unless bone metastases are suspected.
- Concomitant medications/treatments: Record the concomitant medication timely;
- Adverse events: Observe and record adverse events during the study at any time. The recording of adverse reactions should start from the first day of study treatment until at least 28 days after the last treatment, or until all serious or drug-related toxicities are safely recovered to NCI-CTC-AE 5.0 standard grade 1 or below;
- Drug distribution/recovery: Corresponding records are required to be made.
- Patient compliance: Calculate On days 14 and 28 of cycle 1, and on day 28 of each cycle thereafter, the dose of medication taken, counts, and adherence were calculated and recorded in the eCRF.

7.3 End-of-treatment/withdrawal from study

Subjects would continue medication until disease progression, intolerable toxicity, withdrawal of informed consent, or discontinuation of medication at the discretion of the investigator. At the

end of study treatment or withdrawal from the study, if the following examinations (except echocardiography, tumor imaging evaluation) should be done unless have been completed within 7 days before:

- ECOG PS
- Vital signs,
- Physical examination;
- Blood routine;
- Urine routine;
- Stool routine;
- Blood biochemical test;
- Pregnancy test;
- 12-Lead ECG;
- Echocardiography;
- Tumor imaging evaluation (if not done within 4 weeks);
- Concomitant medications/treatments;
- Adverse events;
- Drug recovery: recovered the remaining medicine.

7.4 Follow-up

The subject will enter the follow-up period from the day after the last medication, and the following follow-up should be carried out until the completion of the survival follow-up (subjects who have not been enrolled in the group do not need to undergo follow-up), or 24 months after the last subject is enrolled, or when the investigator considers necessary to end the trial early:

- Safety follow-up: Within at least 28 days after the last study medication, the investigator can select blood routine, blood biochemistry, electrocardiogram, echocardiography, physical examination, vital signs, ECOG score and other clinical evaluation measures according to the specific conditions of the subjects, and record as unscheduled examination. Drug-related adverse events should be followed up until disappearance, remission to baseline level or ≤ grade 1, stable status, or reasonable explanation (whichever comes first);
- Efficacy follow-up: Subjects exit for reasons except PD and death need to be followed up for efficacy. Tumor imaging evaluation should be performed at least once every 12 weeks (±7 days) from the last tumor imaging evaluation during the study period, until disease progression, initiation of other anti-tumor drugs or death (whichever comes first), and detailed record of date and tumor imaging evaluation results of each follow-up should be made;
- Survival follow-up: All non-dead subjects who complete safety follow-up and efficacy follow-up (whichever completed later) will be required to undergo survival follow-up. From the date of completing the safety follow-up and efficacy follow-up (whichever completed later), survival information (date of death and cause of death) will be collected every 12 weeks (± 7

days) at least once every 12 weeks (± 7 days) by telephone inquiries or clinical follow-up of the subjects, their family members or local physicians until death, loss to follow-up, or end of OS data collection, whichever occurs first. The details of each survival follow-up should be recorded and entered in the corresponding eCRF form.

8 Efficacy Assessment

8.1 Imaging evaluation

High resolution enhanced CT or enhanced MRI is recommended for tumor imaging evaluation. Subjects with a history of contrast hypersensitivity were managed according to the trial center's guidelines for the prevention of contrast hypersensitivity reactions to either contrast-enhanced CT or contrast-enhanced MRI when possible. If the subject is strictly contraindicated to contrast media, a plain CT scan is permitted.

Imaging evaluation in the screening period will focus on breast, chest and abdome, and craniocerebral, neck, and pelvic when clinical indications show it is necessary. The results of CT/MRI evaluation within 14 days before signing the informed consent (and within 4 weeks before the first dose) can be used as long as they conform with all other requirements of this protocol.

Imaging examinations during the trial period should be performed under the same conditions as the baseline examinations (contrast agent use, etc.). The time points of tumor imaging during dosing were determined from the start of study treatment, regardless of the time during which dosing was suspended. The allowable time window for tumor imaging is ±7 days, and the specific evaluation time points are as follows

- First evaluation will be conducted on Day 28 of Cycle 2, and every 2 cycles for first 18 cycles, and every 3 cycles thereafter;
- The first PR/CR should be confirmed after 4 weeks (before the next efficacy assessment as specified by the protocol), and the evaluation for confirming would not change the time points fixed before;
- Perform tumor assessment at the end of treatment or subject withdrawal (if not done within the previous 4 weeks).

According to RECIST 1.1 criteria, bone scan and PET are not suitable for target lesion assessment. If these tests are used to assess non-target lesions, the frequency of assessment of these non-target lesions can be reduced. For example, bone scans can be repeated only when CR is confirmed in target lesions or when bone lesions are suspected to have progressed.

The imaging evaluation of this trial will be performed at site (on site review) by an experienced, qualified study physician designated by site.

Response evaluation based on tumor imaging was performed according to RECIST 1.1 criteria. The final evaluation result is subject to the investigator's evaluation.

8.2 Primary endpoint

Objective response rate (ORR): Refers to the percentage of subjects with the best response of CR or PR in the total efficacy analysis set during the period from the start of subjects receiving the treatment regimen to the progression. Objective tumor response was assessed using Response Evaluation Criteria in Solid Tumors (RECIST 1.1 criteria). Subjects must have measurable tumor lesions at baseline. According to RECIST 1.1, the efficacy evaluation criteria include: complete remission (CR), partial remission (PR), stable disease (SD, stable disease ≥ 24 weeks), and disease progression (PD).

8.3 Secondary endpoints

Progression free survival (PFS): Defined as the time from the start of the subject receiving study treatment to the first imaging confirmed disease progression (PD) or death from any reason. If the subject does not develop PD or survives at the end of the study, the cut-off time shall be the time of the last efficacy evaluation before the cut-off date. Second progression or death after radiotherapy in patients with solitary intracranial progression would be counted as PFS2 event that analyzed in patients with BM.

Overall survival (OS): Refers to the time period from the date of receiving study treatment to the date of death (any cause). Subjects without an observed death will be censored at the time of confirmed survival. The OS for subjects censored is defined as the time from treatment to be censored.

Secondary endpoints would be evaluated according to RECIST 1.1 criteria, the analysis included study treatment and tumor evaluation during follow-up. If a patient has several evaluations that can be determined as PD, the first evaluations will be used in ORR analysis. Recurrence, new lesions, or death were considered to have reached the study end point, and patients would also be considered to have progressed with other systemic or observed target antitumor therapy.

9 Safety Assessment

During the trial period, the safety of the study drug will be evaluated through adverse event records (including serious adverse events), laboratory tests, vital signs, physical examination, ECOG score, echocardiography and electrocardiogram records. During the trial, the symptoms and signs of the subjects after the drug should be closely observed. Adverse events/reactions that occur should be dealt with in a timely and effective manner to ensure the safety and interests of the subjects. After the occurrence of adverse drug events/reactions has been dealt with in a timely and effective manner, the type, symptoms, time of occurrence, degree (or grade), symptomatic treatment methods and outcomes should be recorded, and then the adverse events should be analyzed, assessed, and counted as the basis for the continuation of the study.

9.1 Physical examination and vital signs

The physical examination is carried out by the research doctor, and including evaluation of head, skin, lymph nodes, eyes, ears, nose, throat, oral cavity, respiratory system, cardiovascular

system, abdomen, genitourinary system, musculoskeletal system, nervous system and mental state.

Vital signs include the following: body temperature, blood pressure, respiratory rate, and heart rate.

The ECOG PS was scored by theresearch doctor according to Appendix 2.

9.2 Laboratory test

The samples of laboratory test will be collected at the time points specified in the "Flow chart". The following laboratory indicators shown in table 6 will be evaluated by investigators. Unscheduled clinical laboratory tests may be performed at any time for subjects' safety.

Table 6 Items requirements for laboratory tests

Blood routine	Blood chemistry	Urine routine	Stool routine	Infectious disease screening[c]	Other
RBC, WBC, PLT, Hb, neutrophil, LC	TBIL, DBIL<br>IBIL, ALT, AST, AKP, γGT, LDH, total protein, albumin, BUN, Cr, uric acid, blood glucose, TG, cholesterol, potassium, sodium, chloride, calcium, phosphorus, magnesium	urine red blood cells, urine white blood cells, urine glucose,<br>urine protein[a]	fecal occult blood test	five indexes of hepatitis B, HCV antibody,<br>HIV antibody	Pregnancy test[b]

a) When urine protein test shows ++ or above, perform 24-hour urine protein quantification test.

b) Women of reproductive age should be tested for blood HCG during screening and at the end/exit of treatment to exclude pregnancy, and urine HCG at other time points.

c) If the hepatitis B and C test results are abnormal, the investigator should determine whether to perform the virus replication (HBV-DNA, HCV-RNA) test.

9.3 ECG

The 12-lead ECG will be performed by a qualified physician according to the time points specified in the clinical trial flow chart. All ECG examinations are required to be performed at least 10 minutes after the subject has rested in a quiet recumbent position. Contents include at least: heart rate, QT, QTc and P-R interval. During the screening period, three examinations (at least 10 minutes apart) are required, and the mean of the three QTcFs will be used as the baseline QTcF.

To assess subject safety, study physicians will compare ECG results to baseline. If the QTc interval increases by >30 msec from the baseline, or the absolute value of the QTc interval is ≥ 470ms, 2 additional ECG examinations are required, with an interval of at least 10 minutes, to determine the accuracy of the original measurement and exclude the abnormal ECG result caused by incorrect routing of the leads. If the machine-read QTc value is prolonged, repeat measurements may not be performed if a qualified physician determines that the QTc value is within an acceptable range, as described above.

9.4 Echocardiography

Echocardiography will be performed by a qualified physician according to the time points

specified in the clinical trial flow chart. LVEF will be assessed and monitored by the study physician at protocol-specified time points during administration of the investigational drug. During the administration process, according to the judgment of the investigator, subjects with symptoms of heart failure or clinically significant LVEF decline should be treated and monitored in accordance with standard medical guidelines, and a cardiologist should be consulted if necessary. In the event of clinically uncontrollable symptoms of severe heart failure (NYHA class III or IV) or a significant decrease in LVEF (below the lower limit of normal or below 50%), administration of the trial drug should be discontinued as specified in Section 5.4 - Dose Adjustment Protocol medication, and continue to be treated and monitored according to standard medical guidelines.

9.5 Adverse event (AE)

9.5.1 Definition of AEs

Adverse events refer to any adverse medical events that occur after the subjects of the clinical trial signed the informed consent, but the events do not necessarily have a causal relationship with the study drug. An adverse event can be any unexpected, unfavorable symptom, sign, disease, or abnormal examination result, whether related to the study drug or not. Adverse events include the following: 1) a medical condition/disease that existed prior to initiation of study treatment, and was only recorded as an adverse event if it worsened after initiation of study drug; 2) any new adverse event; 3) experimental Abnormal changes in laboratory findings that are considered clinically significant constitute an adverse event.

The investigator shall record in detail any adverse events that subjects have experienced, including the name of the adverse event and description of all relevant symptoms, occurrence time, severity, relevance with the test drug, duration, measures taken, and final results and outcomes.

9.5.2 Grades of AEs

Refer to the grading standard for adverse drug events in NCI-CTCAE 5.0 version. AEs not listed in NCI-CTCAE 5.0 version can refer to the following standards:

Grade 1: Mild; asymptomatic or mild symptoms; clinical or diagnostic observations only; intervention not indicated.

Grade 2: Moderate; minimal, local or noninvasive intervention indicated; limiting age-appropriate instrumental activities of daily living (ADL), which refer to preparing meals, shopping, using the telephone, managing money, etc.

Grade 3: Severe or medically significant but not immediately life-threatening; hospitalization or prolongation of hospitalization indicated; disabling; limiting self-care ADL, which refer to bathing, dressing and undressing, feeding self, using the toilet, taking medication, and not bedridden.

Grade 4: Life-threatening consequences; urgent intervention indicated.

Grade 5: Death related to AE.

9.5.3 Judgment criteria for the causal association between adverse events and test drugs

Regardless of whether it is related to the investigational product, or even the investigational product has not been accepted, all unexpected clinical manifestations after signing the informed consent form should be reported as AEs. Any adverse reactions complained by subjects and abnormal laboratory test results during treatment should be truthfully recorded. In addition, indicate the severity, duration, management, outcome, and concomitant medication/treatment of the AE. Investigators should comprehensively evaluate the relationship between AEs and investigational product. The causality can be divided into five categories, which are definitely related, possibly related, possibly not related, definitely not related, and unassessable. "Definitely related", "possibly related" and "unassessable" are all listed as drug-related adverse reactions.

9.6 Serious adverse events (SAE)

9.6.1 Definition of serious adverse event

Serious adverse event (SAE) refers to a medical event that requires hospitalization or prolonged hospitalization, causes disability, affects workability, is life-threatening, causes death, and causes congenital anomaly. Adverse events that meet one or more of the following criteria are SAE:

- Results in death;
- Is life-threatening (refers to an event in which the patient is at risk of death at the time of the event);
- Requires inpatient hospitalization or causes prolongation of existing hospitalization;
- Results in persistent or significant disability/incapacity;
- May have caused a congenital anomaly/birth defect;
- Other important medical event means an adverse event that may not be immediately life-threatening or result in death or hospitalization but may jeopardize the subject or require medical or surgical intervention to prevent one of the outcomes listed.

9.6.2 Abnormality of liver function test

If AST and/or ALT levels are abnormal and total bilirubin levels are abnormally elevated, and the following conditions (1), (2) and (3) are met and there is no other cause of the abnormality.

Conditions	Criterion
(1) Abnormality of ALT or AST	With normal baseline value: ALT/AST ≥ 3×ULN during the trial;<br>Without normal baseline value: ALT/AST ≥ two times of baseline value, and ≥ 3× ULN; or absolute value ≥ 8× ULN during the trial
(2) Abnormality of TBIL	With normal baseline value: TBIL > 2 × ULN during the trial;<br>Without normal baseline value: TBIL increased by ≥ 1×ULN or absolute value > 3×ULN during the trial
(3) ALP < 2 × ULN (or unavailable) , without hemolysis	

If subjects have abnormal AST and ALT levels and abnormally elevated TBIL levels during treatment or follow-up, they should return to the study site for evaluation as soon as possible (preferably within 48 hours) after receiving the abnormal results. The assessment should include laboratory tests, detailed history and physical evaluation, and should consider the possibility of liver tumors (primary or secondary).

In addition to repeated detection of AST and ALT, laboratory tests should also include albumin, creatine kinase, total bilirubin, direct and indirect bilirubin, gamma-glutamyltransferase,

prothrombin time [PT] / International normalized ratio [INR]), alkaline phosphatase, etc. Further tests may include testing for acute hepatitis A, B, C, and E and liver imaging (e.g., biliary tract).

Detailed medical history should include alcohol use, acetaminophen, soft drugs, supplements, family history, occupational exposure, sexual behavior, travel history, contact with patients with jaundice, surgery, blood transfusion, liver disease, or allergic disease. If repeated testing confirms compliance with the above standard, the possibility of abnormal liver function should be considered in the absence of other causes of abnormal liver enzymes without waiting for all liver function etiological test results. Such abnormal liver function tests should be reported as SAE.

9.6.3 Disease progression and death

Disease progression is defined as the deterioration of the condition of the subjects caused by the study indication, including the progress confirmed by imaging and clinical symptoms and signs. Progression of disease is considered to be a new metastasis of the primary tumor or progression of the original metastasis. Life-threatening events, events requiring hospitalization or prolonged hospitalization, events resulting in permanent or severe disability/disability/inability to work, congenital abnormalities or birth defects which is resulted from symptoms and signs of disease progression, is not reported as SAE. If there is any uncertainty as to whether SAE is due to disease progression, it should be reported as SAE.

In the study population, "disease progression" is expected to occur and should not be reported as an AE term. When disease progression occurs, the event used to confirm disease progression should be reported as AE. For example, if a subject has a seizure that is determined to be associated with brain metastases, the AE term should be recorded as "seizure" rather than "disease progression" or "brain metastases".

If a subject dies during the trial, regardless of whether she has received new antitumor therapy, this must be reported as SAE (see Table 7 AE/SAE collection and follow-up period guidelines). Deaths assessed by the investigator as possibly due to signs and symptoms of disease progression should be recorded in the eCRF and reported as an SAE. The term "death" shall not be used as an AE or SAE term, but as a result of an event, and the event that caused or resulted in the death shall be recorded as AE or SAE. If the cause of death is unknown and cannot be determined at the time of reporting, the AE or SAE terminology records an "unexplained death."

9.6.4 Hospitalization

Adverse events that lead to hospitalization or prolonged hospitalization in clinical research should be regarded as SAEs. Any initial admission (including ≤24 hours) meets this criterion.

The following hospitalizations are not SAE:

- Rehabilitation facilities;
- Nursing homes; emergency rooms;
- Day surgery (such as outpatient/ ambulatory surgery);
- Social reasons (medical insurance reimbursement, etc.)

- Hospitalization or lengthening of hospital stay that is not related to worsening AEs is not classified as SAEs, for example:
  - ✓ Hospitalization due to previous diseases; no new AEs; no exacerbations of existing diseases (such as re-examination of persistent laboratory abnormalities);
  - ✓ Hospitalization for health management reasons (such as annual physical examination);
  - ✓ Planned hospitalizations during clinical trials (such as hospitalization as required by the trial protocol);
  - ✓ Elective hospitalization that has nothing to do with worsening adverse events (such as elective cosmetic surgery);
  - ✓ The planned treatment or surgery should be recorded in the trial protocol and/or the subject's baseline data;
  - ✓ Admission to the hospital only for the use of blood products.

Diagnostic or therapeutic invasive (such as surgery) and non-invasive operations are not reported as AEs. However, when the disease or symptom that is the cause of this operation meets the definition of an AE, it should be reported. For example, acute appendicitis that occurs during the reporting period should be reported as an AE, and the appendectomy performed accordingly should be recorded as the management of the AE.

9.6.5 The procedures for SAE reporting

The report of SAEs should start from signing the informed consent form until the end of the safety follow-up. If a SAE occurs during the trial, whether it is the first report or follow-up report, the investigator must immediately fill in the “Serious Adverse Event (SAE) Report Form”, sign and date it. The investigator should notify the main research unit and Hengrui drug safety department, and timely report to the relevant units according to the regulations (Appendix 5).

SAE that occurred after the safety follow-up period should be collected for those suspected to be related to the study drug. SAE should be recorded in detail with symptoms, severity, correlation with the test drug, occurrence time, treatment duration, measures taken, follow-up time and methods, and outcome, etc. If the investigator determines that a SAE is not related to the investigational drug but is potentially related to study conditions (such as discontinuation of the original treatment or comorbidities during the trial), the relationship should be specified in the narrative section of the SAE report form. Follow-up reports should be submitted immediately if the intensity of an ongoing SAE or its relationship to the drug being tested changes. If the investigator believes that the previously reported SAE information has been misreported, it can be corrected, revoked, or degraded in the follow-up report and reported according to the SAE reporting procedures.

The email address for hengrui Drug Safety Department to receive SAE report of this project is: hengrui_drug_safety@hrglobe.cn。

9.6.6 Pregnancy

Pregnant subjects meet the exclusion criteria. The investigator must report to Hengrui within

24 hours of knowing the pregnancy and fill in the "Hengrui Clinical Trial Pregnancy Report/Follow-up Form" at the same time.

The mother and the fetus must be followed up at least until the birth of the infant and one month after the birth of the infant. And report the results to Hengrui.

If the subject experiences SAE at the same time during pregnancy, the SAE Report Form should be completed and reported within the time limit and requirements of SAE.

If a pregnancy results in an abnormal outcome (stillbirth, spontaneous abortion, defect/ congenital anomaly), this must be reported as an SAE. At the same time.

9.6.7 Other antitumor treatments

SAEs were recorded from the time subjects signed the informed consent until the end of the safety follow-up period (28 days after the last use of the study drug). If subjects initiate additional antitumor therapy before the end of the safety follow-up period, the reporting period for non-fatal SAE will expire until the initiation of new antitumor therapy, unless the SAEs suspected to be related to the study drug. If the death occurred during the safety follow-up period, regardless of whether the subject received other treatment, it must be reported as a SAE.

9.7 Follow-up of AE/SAE

All AEs/SAEs should be followed until the safety follow-up period ends or AEs/SAEs disappear, remission to baseline level or ≤ 1, stable state, or reasonable explanation (e.g., loss of follow-up, death).

The investigator should inquire at each visit about AE/SAE events that have occurred since the last visit and provide follow-up information in a timely manner based on queries received. At the end of the study, the principle of AEs/SAEs collection and follow-up period after the subjects' last medication can refer to table 7 below.

Table 7 Principles of AE/SAE collection and follow-up period

Category	Collection/record	Follow-up
AEs unrelated to study drugs	Until the end of the safety follow-up period or the initiation of new antitumor therapy (whichever comes first)	Until the end of the safety follow-up period
AEs related to study drugs	Until the end of the safety follow-up period	Follow-up to disappearance, remission, or baseline level, or ≤ 1, or stable state, or reasonable explanation (e.g., loss of follow-up, death).
SAEs and SIEs unrelated to study drugs	Until the end of the safety follow-up period or the initiation of new antitumor therapy (whichever comes first) Until the end of the safety follow-up period	Until the end of the safety follow-up period or the initiation of new antitumor therapy (whichever comes first) Until the end of the safety follow-up period
SAEs and SIEs related to study drugs	Indefinitely	Follow-up to disappearance, remission, or baseline level, or ≤ 1, or stable state, or reasonable explanation (e.g., loss of follow-up, death).

10 Clinical Trial Management

10.1 Ethics and Informed Consent

10.1.1 Ethics

This clinical trial must comply with the Declaration of Helsinki (2008 edition), the Good Clinical Practice for Drugs (GCP) promulgated by the NMPA, and related regulations. Prior to the start of the trial, approval from the unit's ethics committee must be obtained before the study can begin. During the clinical research period, any modification of the trial protocol should be reported to the ethics committee and put on record. The principal investigator is responsible for regularly submitting the interim report of the trial in accordance with the relevant requirements of the ethics committee, and should notify the ethics committee that the trial has ended after the trial is over.

10.1.2 Informed consent

Subjects must give informed consent to participate in this trial before receiving the drug in this trial, in order to protect the legitimate rights and interests of the subjects. The researcher is responsible for fully and comprehensively introducing the purpose of this study, the role of the drug, possible toxic and side effects and possible risks to the subjects or their designated representatives. Risks and benefits. Conversation is a very important informed consent process. If the subjects and their legal representatives are illiterate, witnesses should participate in the informed consent process. After the subjects or their legal representatives give oral consent, they should sign the informed consent form. Signed on the same day. The informed consent form should indicate the version number and version date.

10.2 Investigational product management

Assigned a person to be responsible for the management, distribution and recovery of the study drugs. Investigators must ensure that all investigational products are used only for subjects participating in the clinical trial. The dosage and usage of study drugs should follow the trial protocol, and the remaining drugs should be returned to the sponsor. It is forbidden to transfer clinical medications to anyone other than clinical trial participants. CRA is responsible for supervising the supply, use, storage and disposal of remaining drugs.

10.3 Amendment of the plan

Any necessary changes to the protocol must be made in the form of protocol revisions, and must be submitted to the ethics committee for approval or filing after the principal investigator's signature and approval, and the details of previous revisions should be explained in the protocol.

10.4 Quality management

- The clinical research unit must be a drug clinical research base with clinical research conditions as determined by NMPA (former CFDA);
- Investigators must be physicians trained in clinical trials and work under the direction of senior professionals;
- The pre-test inspection clinical ward must meet the standardized requirements to ensure

that the rescue equipment is fully equipped;

- Administration by professional nursing staff, learn about the taking of medicines in detail, and ensure subjects' compliance;
- Each site must strictly follow the research plan and fill in the eCRF truthfully;
- Supervisors should follow standard operating procedures, supervise the conduct of clinical trials, confirm that all data records and reports are correct and complete, that all eCRFs are filled in correctly and are consistent with the original data, and ensure that the trial is carried out in accordance with the clinical research protocol;
- Once a SAE occurs, the supervisors must promptly notify each trial unit, and if necessary, temporarily suspend the study;
- All centers participating in the trial should be audited by the drug regulatory authorities, and it is especially important that the investigators and their related personnel provide convenience and time for monitoring and auditing.

10.5 Data management

10.5.1 Data collection

This study uses eCRF to collect research data. The investigator or specialized data entry personnel (CRC) should enter the data into the eCRF system according to the requirements of the visit process, allowing the researcher or CRC to modify or interpret the data in question. Once the database is locked, the investigator will receive a file copy of the patient data for archiving at the study facility.

10.5.2 Data Management and Quality Control

In order to ensure the authenticity and reliability of clinical trial data and improve the quality of clinical data, clinical supervisors will review the integrity, consistency and accuracy of the trial data in the clinical database in accordance with standard operating procedures during the course of the trial project, and instruct the staff of the research institution to make necessary supplements or corrections. The data manager challenges the investigator or the CRC about the questionable data, and the researcher or CRC must respond to the challenge and make corrections or interpretations of the questionable data, which can be challenged as many times as necessary until the questionable data is resolved. At the end of the trial project, data managers and medical staff will conduct final quality control of all data in the database, and summarize all protocol deviations and protocol violations that occurred during the trial. After the data in the database meets the quality requirements, the database will be locked and the data management personnel will export the data for data analysis by the statistical department.

10.5.3 Data review and monitoring of research institutions

Investigators must maintain source documents for each patient enrolled in the trial, including study medical records and visit records (inpatient or outpatient medical records) that include demographic indicators and medical information, laboratory data, electrocardiograms, and any other

examinations or evaluations the result of. All information on the eCRF must be derived from the original documents in the patient file. The investigator must also keep the informed consent form signed by the patient.

Investigators must verify that all relevant source documents can be reviewed to verify that they are consistent with the eCRF content. Monitoring criteria require 100% monitoring of informed consent obtained, compliance with inclusion/exclusion criteria, documentation of SAEs, and data required for evaluation of all primary and safety measures. Additional checks for consistency of raw data and eCRF were performed in accordance with the trial-specified monitoring plan. Any information on the patient's identity in the original file will not be made public.

10.6 Protocol violation

All requirements specified in the research protocol must be strictly implemented. Any intentional or unintentional deviation or violation of the protocol and GCP principles can be classified as PD or PV. If PD occurs, the investigator or CRA should fill in the protocol violation record, and describe the time of discovery, time of occurrence, course of event, cause, and treatment measures in the record. The record should be signed by the investigator and reported to the ethics committee.

10.7 Data preservation

In order to meet the requirements of the China Food and Drug Administration (CFDA) for the evaluation and supervision of the clinical trial, investigator should agree to preserve all research data, which includes: the original records of the subjects' hospitalization, informed consent, case report forms, detailed records of drug distribution, etc. The research data should be kept by the research institution until 5 years after the end of the clinical trial. The ownership of all data in this clinical study belongs to the main investigators and Henan Cancer Hospital. Unless required by the CFDA, the investigator shall not provide any information to a third party in any form without the written consent of the sponsor.

10.8 Publication policy

All articles and reports related to the trial cannot be published without the consent of the principal investigator and Jiangsu Hengrui Pharmaceuticals Co., Ltd.

11 Data Analysis and Statistical Methods

11.1 Sample size

Based on Simon's two-stage design, the null hypothesis (P0) of a objective response was 50%, and the target ORR (P1) of 70% was set, with a type I error rate of 0·05 ($\alpha = 0·05$) and power of 80% ($\beta = 0·2$), the sample size was calculated to be 37 (23 in the first stage). Considering 10% loss to follow-up, totally 41 subjects would be enrolled.

The basis for setting P0: Phase I dose exploration trial of pyrotinib monotherapy showed that

in HER2+ MBC patients (half of whom had previously received ≤3 line therapy), the overall ORR of the pyrotinib dose groups was 50% and that of the 400mg group was 87.5% (7 of 8 patients). In phase III PHENIX studies of pyrotinib in combination with capecitabine vs capecitabine in patients with HER2+ MBC (approximately 90% previously treated with 1-2 lines), ORR of pyrotinib monotherapy following progression of capecitabine in the double-blind phase was 38.0% (27/71). Subjects in this study were HER2+ MBC who had received advanced treatment ≤1 line in the past, and the line number was higher. Combined with the above data, the conservative ORR of pyrotinib monotherapy for HER2+ MBC was considered as 50%.

The basis for setting P1: In MonarcHER study, ORR of abemaciclib+ trastuzumab and trastuzumab + chemotherapy for third-line or above triple positive breast cancer was the same, 13.9%, indicating that the ORR of anti-HER2 therapy combined with CDK4/6 inhibitor may be similar to that of anti-HER2 combination chemotherapy. ORR of the PHENIX study in the double-blind phase of pyrotinib combined with capecitabine (line 2-3) was 68.6%. According to these data, the ORR of SHR6390 combined with pyrotinib is estimated to reach 70%.

11.2 Statistical Analysis Program

11.2.1 Analysis population

The analysis population includes the full analysis set (FAS), the per-protocol set (PPS), and the safety set (SS).

FAS: Following the intent-to-treat (ITT) principle, including all enrolled patients who received at least one dose of study treatment; will be used for efficacy analysis.

PPS: All patients who meet the trial protocol, have good compliance, have taken at least one cycle of study drugs (excluding patients who have experienced disease progression with substantial evidence after enrolment), have not received prohibited drugs during the trial period, and completed the eCRF requirements. Missing data will not be imputed. The PPS set is preferred for statistical analysis for efficacy.

SS: All enrolled patients who received at least one dose of study treatment and with safety records after administration. This data set is used for safety analysis.

11.2.2 Statistical analysis methods

The data in this study will mainly be summarized using descriptive statistics. Measurement data will be lists as mean, standard deviation, median, maximum value, minimum value, count data and grade data list frequency (composition ratio), rate, and confidence interval.

All statistical analysis will be calculated using SAS 9.2 statistical analysis software programming. All statistical tests are two-sided, P values less than or equal to 0.05 are considered to be statistically significant for the differences tested, and confidence intervals are used at 95% confidence.

1) Basic characteristics of patients

Calculate the mean, standard deviation, median, maximum, minimum value of quantitative data such as age, height, and weight, and list the frequency and percentage of qualitative data such as gender and ECOG score.

2) Efficacy analysis

Primary efficacy measurement ORR will be calculated incidence rate and 95% confidence interval;

Secondary endpoints PFS and OS will be summarized using the Kaplan-Meier method, and the survival curves will be drawn, and the median and 95% confidence interval will be given.

3) Safety analysis

The safety analysis will be done based on the safety set, and will be limited to descriptive statistical summary, which mainly includes the following: basic characteristics of subjects (including sociodemographic information, past medical history and past medication history); incidence of adverse events, the incidence of adverse events of grade 3 and above; changes in vital signs, physical examination, laboratory examinations before and after the drug, and the relationship with the test drug when abnormal changes occur.

12 Case dropout

All subjects who have filled out the informed consent form and are screened to be eligible to enter the trial have the right to withdraw from the clinical trial at any time. No matter when and for any reason, as long as the subjects who have not completed at least one cycle and cannot be evaluated for safety and efficacy, they are dropout cases (except that subjects exit due to disease progression confirmed by clear medical evidence after enrollment). When a subject drops out, the researcher must record the reason for the drop out, complete the assessment items as possible, and fill in the last visit record carefully in eCRFs. For those who dropout due to adverse reactions and are finally judged to be related to the trial drug, the details should be recorded in the eCRF and notified to the investigator. Subjects who withdraw after screening without obtaining a number are not considered dropout cases. If subjects completed a complete cycle and have detailed records, statistical analysis should be performed in the safety evaluation. Subjects who withdraw from the study cannot re-enter the study and their numbers cannot be used again

13 Expected study duration

1) Sep 2019 - Oct 2019: Start-up stage; including study design and budget confirmation, trial data preparation, center agreement signing, participating in research personnel training, etc.

2) Nov 2019 - Apr 2020: Main stage of the study; the first subject is expected to be enrolled in Nov. 2019, the last subject is expected to be enrolled in Apr. 2020, and the follow-up of the last patient is expected to be completed in Apr. 2022 or the investigator considers that the study should be terminated earlier;

3) Apr 2020 - Jul 2022: Data analysis stage; collect data, make statistical analysis, write and publish papers, conclude the thesis and complete the achievement appraisal.

14 References

1. Siegel, R.L., Miller, K.D. &Jemal, A. Cancer statistics, 2018. CA Cancer J Clin 68, 7-30 (2018).
2. Cardoso, F., et al. 1st International consensus guidelines for advanced breast cancer (ABC 1). Breast 21, 242-252 (2012).
3. Goldhirsch, A., et al. Strategies for subtypes--dealing with the diversity of breast cancer: highlights of the St. Gallen International Expert Consensus on the Primary Therapy of Early Breast Cancer 2011. Ann Oncol 22, 1736-1747 (2011).
4. Sainsbury JR, et al. Epidermal-growth-factor receptors and oestrogen receptors in human breast cancer. Lancet 1985; 1:364−366.
5. Ross JS, et al. Breast cancer biomarker and HER2 testing after 10 years of anti-HER2 therapy. Drug News Perspect. 2009,22:93-106.
6. Moasser MM. The oncogene HER2: its signaling and transforming functions and its role in human cancer pathogenesis. Oncogene. 2007;26(45):6469-87.
7. Slamon DJ, Clark GM, Wong SG, et al. Human breast cancer: correlation of relapse and survival with amplication of the Her2/neu oncogene. Science. 1987;235:177–182.
8. Ponde, N., Brandao, M., El-Hachem, G., Werbrouck, E. &Piccart, M. Treatment of advanced HER2-positive breast cancer: 2018 and beyond. Cancer Treat Rev 67, 10-20 (2018).
9. Velasco-Velazquez, M.A., et al. Examining the role of cyclin D1 in breast cancer. Future Oncol 7, 753-765 (2011).
10. Casimiro, M.C., Velasco-Velazquez, M., Aguirre-Alvarado, C. &Pestell, R.G. Overview of cyclins D1 function in cancer and the CDK inhibitor landscape: past and present. Expert OpinInvestig Drugs 23, 295-304 (2014).
11. Finn, R.S., et al. Palbociclib and Letrozole in Advanced Breast Cancer. N Engl J Med 375, 1925-1936 (2016).
12. Hortobagyi, G.N., et al. Ribociclib as First-Line Therapy for HR-Positive, Advanced Breast Cancer. N Engl J Med 375, 1738-1748 (2016).
13. Goetz, M.P., et al. MONARCH 3: AbemaciclibAs Initial Therapy for Advanced Breast Cancer. J ClinOncol 35, 3638-3646 (2017).
14. Leary AF, Drury S, Detre S, et al. Lapatinib restores hormone sensitivity with differential effects on estrogen receptor signaling in cell models of human epidermal growth factor receptor 2-negative breast cancer with acquired endocrine resistance. Clin Cancer Res 2010; 16: 1486–97
15. Witkiewicz A, et al. Presented at IMPAKT 2014, Brussels, Belgium
16. Gianni L, Bisagni G , Colleoni M , et al. Neoadjuvant treatment with trastuzumab and pertuzumab

plus palbociclib and fulvestrant in HER2-positive, ER-positive breast cancer (NA-PHER2): an exploratory, open-label, phase 2 study[J]. The Lancet Oncology, 2018:S1470204518300019.

17. Witkiewicz, A.K., et al. CDK4/6 inhibition provides a potent adjunct to Her2-targeted therapies in preclinical breast cancer models. Genes Cancer, 5, 261 （2014）.
18. Goel, S., et al. Overcoming Therapeutic Resistance in HER2-Positive Breast Cancers with CDK4/6 Inhibitors. Cancer Cell 29, 255-269 (2016).
19. Witkiewicz, A.K., et al. CDK4/6 inhibition provides a potent adjunct to Her2-targeted therapies in preclinical breast cancer models. Genes Cancer, 5, 261 （2014）.
20. DeMichele, A., et al. CDK 4/6 Inhibitor Palbociclib (PD0332991) in Rb+ Advanced Breast Cancer: Phase II Activity, Safety, and Predictive Biomarker Assessment. Clin. Cancer Res. 21, 995–1001 （2015）.
21. Fujiwara, Y., et al. Phase 1 study of abemaciclib, an inhibitor of CDK 4 and 6, as a single agent for Japanese patients with advanced cancer. Cancer Chemother. Pharmacol. 78, 281–288 （2016）.
22. Patnaik, A., et al. Efficacy and Safety of Abemaciclib, an Inhibitor of CDK4 and CDK6, for Patients with Breast Cancer, Non-Small Cell Lung Cancer, and Other Solid Tumors. Cancer Discov. 6, 740–753 （2016）.
