# Supplementary material for: Dalpiciclib and pyrotinib in women with HER2-positive advanced breast cancer: a single-arm phase II trial
Source: Nat Commun. 2023 Oct 7;14:6272. doi: 10.1038/s41467-023-41955-7 (PMC10560297; doi:10.1038/s41467-023-41955-7)
Supplement: Supplementary file 2 — Reporting Summary [file 41467_2023_41955_MOESM2_ESM.pdf]

nature portfolio

Corresponding author(s): Min Yan

Last updated by author(s): 2023-8-31

Reporting Summary

Nature Portfolio wishes to improve the reproducibility of the work that we publish. This form provides structure for consistency and transparency in reporting. For further information on Nature Portfolio policies, see our Editorial Policies and the Editorial Policy Checklist.

Statistics

For all statistical analyses, confirm that the following items are present in the figure legend, table legend, main text, or Methods section.

n/a	Confirmed	
☐	☒	The exact sample size (n) for each experimental group/condition, given as a discrete number and unit of measurement
☒	☐	A statement on whether measurements were taken from distinct samples or whether the same sample was measured repeatedly
☐	☒	The statistical test(s) used AND whether they are one- or two-sided<br>*Only common tests should be described solely by name; describe more complex techniques in the Methods section.*
☒	☐	A description of all covariates tested
☒	☐	A description of any assumptions or corrections, such as tests of normality and adjustment for multiple comparisons
☐	☒	A full description of the statistical parameters including central tendency (e.g. means) or other basic estimates (e.g. regression coefficient) AND variation (e.g. standard deviation) or associated estimates of uncertainty (e.g. confidence intervals)
☒	☐	For null hypothesis testing, the test statistic (e.g. F, t, r) with confidence intervals, effect sizes, degrees of freedom and P value noted<br>*Give P values as exact values whenever suitable.*
☒	☐	For Bayesian analysis, information on the choice of priors and Markov chain Monte Carlo settings
☒	☐	For hierarchical and complex designs, identification of the appropriate level for tests and full reporting of outcomes
☒	☐	Estimates of effect sizes (e.g. Cohen's d, Pearson's r), indicating how they were calculated

Our web collection on statistics for biologists contains articles on many of the points above.

Software and code

Policy information about availability of computer code

Data collection	Data were collected using Electronic Data Capture System..
Data analysis	All statistical analysis were performed with SAS version 9.2.

For manuscripts utilizing custom algorithms or software that are central to the research but not yet described in published literature, software must be made available to editors and reviewers. We strongly encourage code deposition in a community repository (e.g. GitHub). See the Nature Portfolio guidelines for submitting code & software for further information.

Data

Policy information about availability of data

All manuscripts must include a data availability statement. This statement should provide the following information, where applicable:

- Accession codes, unique identifiers, or web links for publicly available datasets
- A description of any restrictions on data availability
- For clinical datasets or third party data, please ensure that the statement adheres to our policy

The raw clinical data are protected and are not available due to data privacy laws. The de-identified datasets supporting the findings of this study are available for academic purposes on request from the corresponding author, Min Yan (ym200678@126.com) for 5 years, with the approval of the Institutional Ethical Committees. The trial protocol is available as Supplementary Note in the Supplementary Information. Source data are provided with this paper. The remaining data are available within the Article and Supplementary Information.

Human research participants

Policy information about studies involving human research participants and Sex and Gender in Research.

Reporting on sex and gender	41 female adult patients were enrolled in this study.
Population characteristics	Female patients aged 18 to 70 yrs with histologically confirmed advanced HER2-positive breast cancer, previously treated with no more than one systemic therapy in advanced setting, not suitable for or rejecting chemotherapy, were eligible for this trial. Further eligibility criteria included at least one measurable lesion based on Response Evaluation Criteria in Solid Tumors (RECIST) version 1.1, an Eastern Cooperative Oncology Group (ECOG) performance status of 0 or 1, a life expectancy of at least 12 weeks, and adequate organ function. Prior trastuzumab was allowed. Women with childbearing potential must have a negative pregnancy test at screening.
Recruitment	Between April 9, 2020, and May 19, 2021, a total of 42 sequential patients from the study site, diagnosed with HER2-positive advanced breast cancer and previously treated with no more than one systemic therapy in advanced settings were screened according to eligibility criteria pre-specified in the study protocol. Written informed consent was obtained from all patients. Totally 41 patients were enrolled and received study treatment. There was no potential self-selection bias.
Ethics oversight	The study protocol and all amendments were reviewed and approved by Ethics Committees from Henan Cancer Hospital. This study was conducted in accordance with International Conference on Harmonization Good Clinical Practice Guidelines (ICH GCP) and Declaration of Helsinki.

Note that full information on the approval of the study protocol must also be provided in the manuscript.

Field-specific reporting

Please select the one below that is the best fit for your research. If you are not sure, read the appropriate sections before making your selection.

☒ Life sciences ☐ Behavioural & social sciences ☐ Ecological, evolutionary & environmental sciences

For a reference copy of the document with all sections, see nature.com/documents/nr-reporting-summary-flat.pdf

Life sciences study design

All studies must disclose on these points even when the disclosure is negative.

Sample size	A Simon minimax two-stage design was used to estimate the sample size, with one-sided alpha of 5% and power of 80%. The null hypothesis (P0) was set as an objective response rate (ORR) of 50%, the expected ORR (P1) was set as 70%. Accordingly, the first stage was planned to enroll 23 patients. If more than 12 of these patients achieved responses, 14 additional patients would be recruited in stage II. The study was deemed to meet its primary endpoints if confirmed responses were observed in more than 23 patients out of a total of 37 response-evaluable patients. Considering a dropout rate of 10%, totally no more than 41 patients would be enrolled.
Data exclusions	According to the study protocol, efficacy analyses were performed in eligible participants who received at least one efficacy evaluation, and safety analyses were carried out in all patients who received at least one dose of study treatment.<br>One patient's updated information showed she had a HER2 immunohistochemistry (IHC) score of 1+ and HER2 gene amplification by fluorescence in-situ hybridization (FISH), which did not meet the inclusion criteria. Thus, she was excluded from the efficacy analysis though she received study treatment.
Replication	Biomarker analysis was not done due to insufficient materials. Replication was not applicable to our clinical study.
Randomization	As this was a single-arm study, no randomization was performed.
Blinding	As this was a single-arm, open-label trial, blinding was not applicable.

Reporting for specific materials, systems and methods

We require information from authors about some types of materials, experimental systems and methods used in many studies. Here, indicate whether each material, system or method listed is relevant to your study. If you are not sure if a list item applies to your research, read the appropriate section before selecting a response.

Materials & experimental systems

n/a	Involved in the study	
☒	☐	Antibodies
☒	☐	Eukaryotic cell lines
☒	☐	Palaeontology and archaeology
☒	☐	Animals and other organisms
☐	☒	Clinical data
☒	☐	Dual use research of concern

Methods

n/a	Involved in the study	
☒	☐	ChIP-seq
☒	☐	Flow cytometry
☒	☐	MRI-based neuroimaging

Clinical data

Policy information about clinical studies

All manuscripts should comply with the ICMJE guidelines for publication of clinical research and a completed CONSORT checklist must be included with all submissions.

Clinical trial registration	NCT04293276
Study protocol	The study protocol is available in the Supplementary Information file.
Data collection	41 patients from Henan Cancer Hospital were enrolled and started study treatment between April 9, 2020 and May 19, 2021. All efficacy and safety data were collected at the study site.
Outcomes	The primary endpoint was objective response rate (the proportion of patients with a best overall response of complete or partial response). Secondary endpoints included progression-free survival (defined as the time from the first dose of study treatment to the documented disease progression or death due to any cause, whichever occurred first; second progression or death after radiotherapy in patients with solitary intracranial progression would be counted as PFS2 event that analyzed in patients with BM), overall survival (defined as the time from the first dose of study treatment to death due to any cause), and safety.
